# A perspective on tumor radiation resistance following high-LET radiation treatment

**DOI:** 10.1007/s00432-024-05757-8

**Published:** 2024-05-02

**Authors:** Yogendra Singh Rajpurohit, Dhirendra Kumar Sharma, Mitu Lal, Ishu Soni

**Affiliations:** 1 Molecular Biology Division, Bhabha Atomic Research Centre, 2-46-S, Modular Lab, A-Block, Mumbai, 400085 India; 2 Homi Bhabha National Institute, DAE- Deemed University, Mumbai, 400094 India

**Keywords:** Linear energy transfer (LET), Photon therapy, Relative biological effectiveness (RBE), Clustered DNA lesions, Translesion synthesis (TLS)

## Abstract

High-linear energy transfer (LET) radiation is a promising alternative to conventional low-LET radiation for therapeutic gain against cancer owing to its ability to induce complex and clustered DNA lesions. However, the development of radiation resistance poses a significant barrier. The potential molecular mechanisms that could confer resistance development are translesion synthesis (TLS), replication gap suppression (RGS) mechanisms, autophagy, epithelial-mesenchymal transition (EMT) activation, release of exosomes, and epigenetic changes. This article will discuss various types of complex clustered DNA damage, their repair mechanisms, mutagenic potential, and the development of radiation resistance strategies. Furthermore, it highlights the importance of careful consideration and patient selection when employing high-LET radiotherapy in clinical settings.

## Overview

Cancer emerges as a worldwide health emergency, standing as a primary cause of both mortality and morbidity across the human population (Ferlay 2020). According to the World Health Organization (WHO), in 2020, there were approximately 18.1 million new cancer cases, resulting in 10 million deaths. The WHO predicts a staggering increase in cancer cases over the next two decades, estimating around 28 million cases by 2040. Furthermore, projections indicate 16.3 million deaths, marking a 63.7% increase from the statistics recorded in 2020 (Sung et al. 2021). Thus, these findings and estimates highlight the urgent need for effective cancer treatment strategies. A range of treatment modalities, such as surgery, radiation therapy (RT), chemotherapy, immunotherapy, targeted therapy, stem cell transplantation, natural antioxidants, nanoparticles, ablation therapy, radionics, chemodynamic therapy, ferroptosis and sonodynamic therapy, as well as multidisciplinary approaches, are utilized in the battle against cancer (Debela et al. 2021; Pucci et al. 2019; Arruebo et al. 2011; Moo et al. 2018). Among these treatments, RT is a potent method, especially when combined with other treatment modalities. In principal, RT should be effective against all types of tumor cells. However, individual cells within tumors exhibit varying degrees of sensitivity to radiation, resulting in diverse treatment outcomes (Wang et al. 2018; Baskar et al. 2012).

Biological responses triggered by radiation exposure depend on energy deposition per unit length of radiation track (keV/μm) known as linear energy transfer (LET), along with the cellular and tissue water activity and the composition of the extracellular matrix (ECM). Low-LET radiation (γ-rays and X-rays) is termed "sparsely ionizing" because it causes scattered ionizing events in various directions across space. In contrast, high-LET radiation, exemplified by protons (≤ 50 keV/µm), carbon ions (C-ions) (≤ 200 keV/µm), and alpha-emitting radionuclides (60 to 110 keV/μm), is referred to as "densely ionizing" because its ionizing effect predominantly occurs in the narrow region at the end of the primary path (Bragg peak). This results in more complex and clustered DNA damage, characterized by the presence of more than two lesions per DNA helical turn, including strand breaks, base damage, and abasic sites (Mladenova, et al. 2022; Timm et al. 2018; Kruijff et al. 2015). This feature contributes to its greater relative biological effectiveness (RBE) in causing DNA damage and cell death (Paganetti et al. 2002; Oden et al. 2017; Ray et al. 2018; Busato et al. 2022). Modern therapy techniques utilize this principle by employing spread-out Bragg peak (SOBP) (Lin et al. 2018; Akagi et al. 2023; Darafsheh et al. 2021). This approach involves employing multiple charged particle beams with partly overlapping bragg peaks, allowing for the precise delivery of effective doses to the tumor in a three-dimensional fashion. This method reduces harm to healthy tissue situated before and after the tumor, in contrast to conventional low-LET photon therapy (γ-rays and X-rays) (Ray et al. 2018).

High-LET radiation induces dense ionization events that result in a substantial formation of complex DNA damage including lethal lesions such as base alterations, single-strand breaks (SSBs), apurinic/apyrimidinic site (AP sites), double-strand breaks (DSBs), and chromosomal aberrations (Nikjoo et al. 1998; Watanabe et al. 2015; Wilkinson et al. 2023). These lesions, collectively referred to as "clustered lesions," occur in very close proximity, typically within a few nanometers (Hada and Georgakilas 2008; Sage and Shikazono 2017). Among all types of damage, clustered lesions present a significant challenge for DNA repair machinery of the cell and explain why high-LET ionizing radiation has higher RBE and highly effective, despite not necessarily causing a greater overall number of DSBs compared to low-LET radiation (Georgakilas et al. 2013; Lorat et al. 2015; Lorat et al. 2016).

Nevertheless, despite progress in radiation techniques and treatment strategies, the emergence of radiation resistance remains a significant obstacle to enhancing treatment outcomes (Busato et al. 2022; Barcellini 2022). Radiation resistance can arise from several sources, including tumor heterogeneity (differences between tumors of identical types in distinct patients, or difference among cancerous cells within a singular tumor, or difference between a primary and a secondary tumor cells), the tumor microenvironment, TLS, RGS, EMT, and epigenetic alterations within cancer cells (Dagogo-Jack and Shaw 2018). Understanding the mechanisms that drive the development of resistance is essential for surmounting this obstacle in both traditional and novel forms of high-LET RT (Sato et al. 2019). Here, we discuss radiation resistance strategies, repair approaches for clustered DNA lesions, and the possible potential for mutagenesis against clustered DNA lesions. Furthermore, we suggest that clinicians exercise the caution when deciding to use high-LET RT as a treatment for tumors instead of low-LET RT, as there may high risk of tumor resistance developing in the medium to longer term.

### Role of DNA damage repair and other molecular mechanisms in radiation resistance against low-LET radiation

Ionizing radiation (IR) exerts a direct impact on the structure of DNA by introducing DNA breaks, specifically DSBs. Additionally, it triggers secondary indirect effects such as the production of reactive oxygen species (ROS), which in turn oxidize proteins and lipids. These ROS also contribute to various forms of DNA damage, including the creation of abasic sites and SSBs (Borrego-Soto et al. 2015). Out of the diverse types of DNA damage caused by radiation therapy, DSBs are particularly harmful (Huang and Zhou 2020; Deng, et al. 2022). There are four primary DNA repair pathways: recombination repair, mismatch repair (MMR), base excision repair (BER), and nucleotide excision repair (NER). Recombination repair deals SSBs and DSBs, while mismatch repair fixes replication errors. BER addresses oxidation-induced base damage and removes AP sites, while NER corrects bulky adducts and cross-linked DNA damage.

Among these, the DSBs repair pathways, namely, nonhomologous end joining (NHEJ) involving DNA-PKs and Ku heteroduplex proteins and homologous recombination (HR) catalyzed by Rad51 and regulated by ATM and ATR kinases, are vital for mending radiation-induced DNA breaks (Dietlein et al. 2014; Dietlein and Reinhardt 2014; Visnes et al. 2018; Zhu et al. 2009). In response to DSBs caused by low-LET radiation, sensor proteins such as Rad24p (Aylon and Kupiec 2003), NBS1/hMRE11/hRAD50 complex (Zhou and Paull 2013), γH2AX (Georgoulis, et al. 2017), Ku (Abbasi et al. 2021), MDC1 (Ruff et al. 2020), and 53BP1 (Noordermeer et al. 2018), initiate downstream signaling responses mediated by sensor kinases (DNA-PKs, ATM, and ATR) (Mladenov et al. 2019a; Mladenov et al. 2019b; Li, et al. 2021; Smith et al. 2010; Goodarzi et al. 2003). Together, this leads to an initiation of the DNA damage response (DDR) triggers a cascade of complex reactions, encompassing cell cycle arrest and DNA repair processes (Marechal and Zou 2013; Cuadrado et al. 2006; Weber and Ryan 2015). In response to low-LET RT, the expression levels of different DNA damage sensors (γH2AX, 53BP1, NBS1, BRCA1/2, and Ku) have the potential to act as predictive biomarkers for assessing the outcomes of RT in cancer patients (Liu, et al. 2020; Stover et al. 2016). Replication protein A (RPA) is a crucial eukaryotic protein involved in binding to ssDNA and has roles in DNA replication, DNA damage repair, replication stress, and cell cycle regulation. RPA may contribute to radiation resistance by utilizing the ATR/Chk1 pathway to alleviate replication stress (Glanzer et al. 2014; Zou et al. 2006). Inhibition of RPA negatively affects the time bound damaged DNA repair, rendering cells radiation-sensitive. XRCC1 (X-ray repair cross-complementing 1), a key BER protein stands out as a significant predictive biomarker for radiation therapy, and mutations within the XRCC1 gene have the potential to heighten sensitivity to ionizing radiation (Hanssen-Bauer et al. 2012; Caldecott 2019; Niu et al. 2013; Eckelmann, et al. 2020). Additionally, p53 gene (a tumor suppressor gene) expression status in cancerous cells profoundly influences the efficacy of RT (Kong et al. 2021).

In addition to DDR mechanisms, poly(ADP-ribosyl)ation (PARylation) and replication stress management are key fundamental processes that play crucial roles in safeguard of genome integrity against DNA damage. PARylation is a posttranslational modification that occurs in response to DNA damage, particularly in nuclear proteins such as histones. It serves to enhance the survival of damaged proliferating cells (Kamaletdinova et al. 2019). PAR is produced from NAD + through the action of Poly (ADP-ribose) Polymerases (PARPs), a group of 18 proteins encoded by different genes (Alemasova and Lavrik 2019). These PARPs are linked to promoting tolerance to RT (Seyedin et al. 2020; Higuchi et al. 2015). After exposure to ionizing radiation, PARP-1 activates the AMPK/mTOR pathway, leading to processes such as autophagy and reduced sensitivity to radiation (Bi et al. 2018; Rose et al. 2020).

Replication stress can arise from various sources, including genome integrity perturbation by clustered DNA damage, conflicts between replication and transcription machinery, and oncogenic stress (Nickoloff et al. 2022). Certain difficult to repair clustered DNA damages activate DNA damage tolerance (DDT) pathways, where specialized polymerases like REV1, POL ζ, POL η, POL κ, and POL i bypass damage to facilitate continuous replication and radioresistance with possibilities to introduce mutations (Chatterjee and Walker 2017). Furthermore, if these underlying issues, such as DNA damage, are not promptly resolved, nucleases facilitate the restart of the fork. Several nucleases, including MUS81 (Marini et al. 2023; Chen et al. 2021), FEN1 (Zhang et al. 2022a; Vaitsiankova et al. 2022; Yang et al. 2022a), EEPD1 (Wu et al. 2015), Artemis (Betous et al. 2018), Metnase, EXO1 (Tomimatsu et al. 2017), DNA2-BLM, MRE11 (Vertemara and Tisi 2023), CtIP (Mozaffari et al. 2021), SLX1-SLX4 (Wyatt et al. 2013; Sarbajna et al. 2014), XPG (Trego et al. 2016), and XPF-ERCC1-SLX4 (Betous et al. 2018; Xu et al. 2021) are implicated in replication stress management and play roles in promoting the repair and restart of replication forks (Nickoloff et al. 2022).

Modulating the cell cycle regulation and distribution by eukaryotic cells is another crucial adaptation for the protection of genome integrity against DNA damage (Morales-Ramirez et al. 2022; Jackson 2002). Upon detecting difficult to repair DNA damage cells may either undergo apoptosis or enter a state of senescence. Conversely, when damage is reparable, cells prolong their cell cycle to facilitate DNA repair and accomplish it. DNA damage induces cell cycle arrest, particularly through the activation of G2/M checkpoints, which are orchestrated by ATM and ATR (Marechal and Zou 2013; Cuadrado et al. 2006; Barnaba and LaRocque 2021; Pai, et al. 2021). These checkpoints play pivotal roles in preventing cells from entering the mitotic phase when DNA integrity is compromised.

Moreover, resistance to low-LET radiotherapy in cancer involves various pathways, such as autophagy (Chaachouay et al. 2011; Chen et al. 2015; Chen et al. 2011; Khan et al. 2020), the NF‐κB pathway (Deorukhkar and Krishnan 2010; Li and Sethi 2010), Akt/cyclin D1/CDK4 signaling pathway (Shimura 2017; Shimura et al. 2011; Shimura et al. 2012), and the Wnt/β‐catenin pathway (Yu et al. 2021; Liu et al. 2022). Furthermore, factors contributing to this resistance encompass tumor genetics and epigenetics, tumor metabolism, the tumor microenvironment, and the presence of nonmalignant cells, including fibroblast-associated cancer cells, tumor-infiltrating lymphocytes, endothelial cells, macrophage-associated cancer cells, and cancer stem cells (Busato et al. 2022; Rycaj and Tang 2014; Peitzsch et al. 2019; Huang et al. 2023; Wu et al. 2023a) (for a more comprehensive understanding, readers are encouraged to consult the cited references). Thus, in a wider perspective, cancer cells have the capability to build resistance against traditional low-LET RT using various mechanisms.

### High-LET radiation causes difficult to repair clustered DNA damage

High-LET radiation produces clustered DNA damage resulting in complex clustered DNA lesion sites (CDS) or multiple damage sites (MDS). These sites include clustered DSBs together with base lesions or AP sites, as well as clusters of non-DSB damage comprising base lesions and ssDNA breaks (Sage and Shikazono 2017; Georgakilas et al. 2013; Goodhead and Nikjoo 1989; Sage and Harrison 2011; Eccles et al. 2011; Sai et al. 2023; Bukowska and Karwowski 2018). At the chromosomal level, chromatin compaction reduces the incidence of DNA damage caused by ionizing radiation (Tang et al. 2019). Nevertheless, repair of high-LET-induced heterochromatic region DSBs take substantial period while DSBs caused by low-LET ionizing radiation are promptly restored within 24 h, irrespective of chromatin compaction status (Lorat et al. 2015; Roobol, et al. 2020). The DNA damages caused by low-LET radiation usually dispersed throughout the genome, and repair mechanisms are highly efficient at addressing such damage (Chatterjee and Walker 2017; Tian et al. 2015). However, repair of CDS and MDS showed slower repair kinetics, resulting in inaccurate repair and chromothripsis (a solitary catastrophic incident results in the extensive reorganization of genetic material within one or few number of chromosomes), or sometimes not at all repaired (Sage and Shikazono 2017; Cortes-Ciriano et al. 2023; Voronina et al. 2020; Kozmin, et al. 2021; Danforth et al. 2022). This distinction is a pivotal reason why high-LET carbon ions exhibit a 2–threefold greater RBE than low-LET ionizing radiation (Okayasu 2012; Allen et al. 2011; Tsujii et al. 2004; Okada et al. 2010; Paganetti 2022). From this viewpoint, the heightened cytotoxicity of high-LET ionizing radiation leverages the innate shortcomings of DNA repair systems when confronted with clustered lesions. This limitation attributed to the absence of natural selective pressure that drives the development of cellular repair systems capable of efficiently repairing clustered DNA damage (Lorat et al. 2015; Asaithamby and Chen 2011). Furthermore, from a clinical perspective, the efficacy of carbon-ion radiation therapy (CIRT) relies less on oxygen compared to low-LET RT (Antonovic et al. 2014; Strigari et al. 2018). As a result, CIRT presents as an efficient therapeutic option for hypoxic tumors, found in melanoma, head and neck cancer, and pancreatic cancer, which typically demonstrate resistance to traditional radiotherapy. (Allen et al. 2011; Kamada et al. 2015; Tinganelli and Durante 2020).

Clustered complex DNA damage can be broadly divided into two primary categories: DSBs and non-DSB oxidative clustered DNA lesions (OCDLs) (Hada and Georgakilas 2008). OCDLs can be further classified into two categories: bistranded clustered lesions (where multiple lesions are closely located on both DNA strands) and tandem clustered lesions (where multiple lesions are closely positioned on the same DNA strand). Several factors influence the repair phenomenon of clustered complex damage, including the type of lesion, lesion proximity, and lesion orientation. Synthetic oligonucleotides have been employed to study OCDL repair with known DNA lesions using mammalian nuclear or whole-cell extracts and purified BER proteins (Bellon et al. 2009; Kazmierczak-Baranska, et al. 2021; Mourgues et al. 2007; Lomax et al. 2004a; Harrison et al. 1999; Eot-Houllier et al. 2005; Boguszewska, et al. 2021; Karwowski 2019, 2021). The repair efficiency of bistranded OCDLs decreases when lesions are within five bases of an AP site or SSB (Mourgues et al. 2007; Lomax et al. 2004b). Lesions such as 8-oxoG, thymine glycol (Tg), and 5,6-dihydrothymine (DHT) reduce repair efficiency and may generate DSBs by replication (Bellon et al. 2009; Lomax et al. 2004a). Repair mechanisms also differ depending on the orientation of the bistranded lesions. In cases where lesions are in the negative orientation (with the opposing strand lesion positioned 5′ to the base opposite the reference lesion), both long and short patch BER processes are engaged. Conversely, in the positive orientation (where the opposing strand lesion is positioned 3′ to the base opposite the reference lesion), short-patch BER is the dominant mechanism (Harrison et al. 1999; Eot-Houllier et al. 2005; Paap et al. 2008). The presence of nearby lesions interferes with the initial phase of the BER pathway, which involves the excision of damaged bases by DNA glycosylases. For instance, an AP site or SSB on opposite DNA strand can significantly hinder the excision of base lesions (Georgakilas et al. 2004). The rate of base lesion excision is compromised when another lesion is nearby, mainly due to interference with glycosylase binding (Harrison et al. 1999; Eot-Houllier et al. 2005; Paap et al. 2008; Singh and Das 2013). Tandem OCDLs, also exhibit compromised repair, similar to bistranded clusters (Venkhataraman et al. 2001; Jiang et al. 2009).

To deal with DSBs generated directly by high-LET radiation tracks or DSBs converted by OCDLs, error-prone processes surpass high-fidelity homologous recombination (HR) repair (Mladenova, et al. 2022). Moreover, increased DNA end resection and DNA binding characteristics of Mre11 and Ku are crucial for repair pathway decisions for complex lesions (Yajima et al. 2013; Averbeck et al. 2014; Hays et al. 2020; Wang et al. 2010). Additionally, it has been suggested that the ATM protein, partners with the Artemis repair protein, plays a pivotal role in addressing complex and "dirty / clustered" DSBs introduced by high-LET radiation (Lobrich and Jeggo 2005; Xue et al. 2009). The results also indicate that ATM reacts differently to DSBs caused by high-LET radiation than to those induced by low-LET radiation. For instance, high-LET radiation significantly reduces ATM-specific phosphorylation of pATF2 and pSMC1. In contrast, the absence of ATM results in a noticeable decrease in the phosphorylation of DNA-PKC at Thr-2609 and adversely impacts the formation of Rad51 foci (Okayasu 2012; Whalen et al. 2008). Together, these findings suggest that the presence of clustered DNA lesions, whether bistranded or tandem, or DSBs, can hinder and/or alter the repair machinery's approach to repairing them, potentially leading to an increased lifetime of these lesions or conversion to more severe DNA lesions such as DSB, which poses a difficult task for the repair machinery.

### Clustered DNA damage implicated by high-LET radiation has high mutagenic potential

The biological ramifications of OCDLs in mammalian cells are complex and influenced by various factors. The damage caused by OCDLs is primarily repaired through BER pathway (Bellon et al. 2009; Mourgues et al. 2007; Byrne et al. 2009; Cunniffe et al. 2014a), and studies have revealed that specific glycosylases, such as MutY, may determine the mutagenic outcome of clustered base damage (Bellon et al. 2009; Kazmierczak-Baranska, et al. 2021; Byrne et al. 2009; Shikazono et al. 2006). Furthermore, bigger risks arise when bistranded / tandem cluster lesions are converted to DSBs. Studies indicate that levels of repair enzymes, DNA replication status, density of non-DSB damage, and the complexities of the BER pathway may dictate whether these lesions are converted into DSBs or undergo repair without such conversion (Sage and Shikazono 2017; Nikitaki et al. 2016). A firefly luciferase reporter assay uncovered that bistranded clusters featuring opposing furans could transition into DSBs within mammalian cells, with the predominant activity observed from the class II AP endonuclease targeting these lesions. In mammalian cells, other bistranded clusters, such as uracil or 8-oxoG, are not converted to DSBs (Malyarchuk et al. 2009). Additionally, there is only scant evidence suggesting that clustered uracil lesions may be converted into DSBs in mammalian cells (Malyarchuk and Harrison 2005). Why bistranded furans clusters are converted to DSBs more frequently than uracil or 8-oxoG lesions is explained by the concept of “passing the baton”. This involves the cooperation of short BER pathway enzymes, such as DNA glycosylase, XRCC1, AP endonuclease (Ape1), DNA pol β, and DNA ligase 1, which interact to transfer repair intermediates to subsequent repair enzymes, preventing the premature release of repair intermediates. However, disruptions to this seamless transfer, or "passing the baton", can occur when dealing with damage types such as clustered furans that necessitate the involvement of long BER pathway instead short BER pathway enzymes (Wilson and Kunkel 2000).

Together, Bistranded / tandem cluster lesions remain unrepaired, they may encounter replication forks during DNA replication, potentially causing replication-induced DSBs or DNA mispairing, which can eventually lead to collapse of replication or incorporation of adaptive mutations (Sage and Shikazono 2017; Sage and Harrison 2011; Eccles et al. 2011; Kozmin, et al. 2021; Cunniffe et al. 2014a; Cunniffe et al. 2014b; Hsu et al. 2022; Naldiga et al. 2020; Shikazono and Akamatsu 2020). These attributes were experimentally validated in proton-irradiated K-rasLA1 mice (Luitel et al. 2018) and high-LET space-irradiation-exposed Bcl2 transgenic C57BL/6 mice (Xie et al. 2020), where it has been observed that a higher incidence of increased carcinoma and DNA replication stress promotes lung carcinogenesis, respectively.

### Does clustered DNA damage introduced by high-LET radiation effectively thwart the radioresistance of cancer cells?

Advancements in research on synthetic clustered DNA lesions have deepened our understanding of the biological impacts linked to these lesions within cellular or tissue environments (Lomax et al. 2004a; Shikazono et al. 2006; Malyarchuk et al. 2004; Malyarchuk et al. 2003; Budworth and Dianov 2003). The clustered DNA lesions exhibit a broad spectrum of diversity and undergo various processing mechanisms influenced by factors such as base modifications, the distance between lesions, and the presence of strand breaks. The mutation spectrum associated with non-DSB clustered lesions encompasses various genetic alterations, including deletions, base substitutions, and insertions/deletions (indels) of 1–2 base pairs, primarily focused on the lesions within these clusters (Mladenova, et al. 2022; Kozmin, et al. 2021; Malyarchuk et al. 2009, 2008). The sequential handling of lesions within a non-DSB cluster results in the formation of SSBs as intermediate repair products. These SSBs can hinder the excision or repair of other lesions within the cluster. Consequently, the repair ability of clustered DNA lesions is diminished, leading to elevated lifetime of these lesions. Notably, the detrimental effects of repair intermediates can be exacerbated during replication, potentially leading to the generation of DSBs from single-stranded DNA gaps (Malyarchuk et al. 2009; Harper et al. 2010; Cannan and Pederson 2016; Kumar et al. 2023; Datta et al. 2005; Wang et al. 2014). While most DSBs caused by low-LET radiation are swiftly repaired during the G1 phase, mainly through the canonical nonhomologous end-joining (cNHEJ) pathway. The role of NHEJ-specific DNA-PKcs in repairing high-LET radiation-induced DSBs in tumor cells has shown (Anderson et al. 2010; Liu et al. 2018). The DSBs caused by high-LET radiation, particularly complex DSBs, exhibit slower repair kinetics and extend throughout the cell cycle and a substantial portion of DSBs undergo repair during the late S-phase and G2-phase through homologous recombination by altering cell cycle checkpoint regulations (Okayasu 2012; Okayasu et al. 2006; Gerelchuluun et al. 2015; Mohammadian Gol et al. 2019). Additionally, complex DSBs repaired slowly and inaccurately through alternative (Alt-EJ) or micro-homology mediated (MMEJ and SSA) DNA end joining, often resulting in deletions ranging from a few base pairs to several hundred bases (Scully et al. 2019; Wang and Xu 2017; Sallmyr and Tomkinson 2018; Hanscom and McVey 2020; Mladenova et al. 2022).

Moreover, high-LET radiation exposure leads to an increased occurrence of ssDNA gaps owing to the clustered pattern of DNA damage. The introduction of persistent ssDNA gaps can trigger cell death pathways. Therefore, many tumor cells have developed mechanisms to avoid and escape ssDNA gaps, such as slowing fork movement (Peng et al. 2018; Cong, et al. 2021), reversing replication forks for repair (Bai, et al. 2020), blocking fork elongation, increasing the presence of RPA, and gaining access to translesion synthesis (TLS) to ensure continuous replication without gaps (Igarashi et al. 2023; Fu et al. 2024; Nayak, et al. 2020; Nayak, et al. 2021; Tonzi and Huang 2019; Saldanha, et al. 2023; Wu et al. 2023b; Li et al. 2019; He et al. 2022; Park et al. 2022; Venkadakrishnan et al. 2023). All of these replication gap suppression (RGS) mechanisms involve distinct players that facilitate ssDNA gap avoidance and may contribute to tumor resistance to high-LET radiation. Recent research has highlighted the role of TLSs in adaptive mutability to evade targeted drugs against EGFR/BRAF (Crisafulli and Siravegna 2023; Russo et al. 2019). The unrepaired complex DSBs lead to cell death, primarily because cells are unable to complete the mitotic process. However, a significant fraction of cells proceed through the cell cycle checkpoint despite the presence of unrepaired clustered DNA lesions, leading to an increased occurrence of chromatid and chromosomal aberrations including breaks in chromatids and chromosomes (Mladenova, et al. 2022; Sadeghi Moghadam et al. 2023). Notably, dynamic movement of DSB repair foci toward less densely packed chromatin, and their aggregation with other repair centers may predispose them to erroneous rejoining, particularly during the late S and G2-phases. Consequently, compared with those exposed to low-LET radiation, cells that survive exposure to high-LET radiation exhibit significant translocations, sister chromatid exchanges, and gross chromosomal abnormalities (Mladenova, et al. 2022; Timm et al. 2018). This leads to more pronounced and intricate reorganization of the chromosomes. Additionally, a study underscores the particular types of DNA damage caused by radiation that evade repair mechanisms, resulting in clustered mutations in germ cells (Adewoye et al. 2015). Thus, ionizing radiation, particularly high-LET radiation, causes additional ssDNA gaps, mutations, and complex clustered DNA damage, leading to the killing of the majority of tumor cells (Du et al. 2022). However, a small fraction of radioresistant tumor cell can survive with increased chromosomal abnormalities and adaptive clustered mutations resulting in radiation resistance against high-LET RT (Sage and Shikazono 2017; Sage and Harrison 2011; Adewoye et al. 2015; Nickoloff et al. 2020).

### High-LET radiation therapy: harnessing its potential with caution

RT stands as an invaluable asset in cancer treatment, providing benefits to approximately half of all cancer patients. The clinical applications of high-LET radiation have seen a worldwide expansion, notably in proton and hadron therapy (Mohamad, et al. 2017; Yang et al. 2018; Hayashi et al. 2019; Tomizawa et al. 2023; Shiba et al. 2022a; Shiba et al. 2023; Musha et al. 2022; Kiseleva, et al. 2022). Despite the increasing utilization of high-LET radiation in clinics, there remains a lack of comprehensive understanding regarding the biological mechanisms underlying its effects. The remarkable feature of high-LET radiation lies in its ability to generate clustered OCDLs sites, and DSBs. The present therapeutic strategy involving the use of high-LET radiation combined with inhibitor therapy has shown initial benefits. Clinical evidence supports its effectiveness in treating various nonsquamous cell histologies, including adenocarcinoma, malignant melanoma, adenoid cystic carcinoma, hepatoma, early-stage and locally advanced non-small cell lung cancer, and bone/soft tissue sarcoma. (Mohamad, et al. 2017; Yang et al. 2018; Hayashi et al. 2019; Tomizawa et al. 2023; Shiba et al. 2022a; Shiba et al. 2023; Musha et al. 2022; Kiseleva, et al. 2022). Synergistic approach of high-LET RT alongside targeted inhibitors of DNA repair pathways (such as BER, HR, and NHEJ) have been adopted (Cesaire et al. 2019; Srivastava et al. 2018; Hirai et al. 2012; Fujisawa et al. 2015; Ma et al. 2015; Bright et al. 2022). Alternatively, targeting immune checkpoint inhibitors (anti-PD-1/PD-L1 and anti-CTLA4) is explored to counteract resistance (Zhang et al. 2022b; Mondini et al. 2020; Bernal, et al. 2024). Furthermore, high-LET RT option considered effective against hypoxic tumors (Kabakov and Yakimova 2021). However, cancer cells adapted to hypoxic conditions, often due to deregulation of hypoxia-inducible factor-1 (HIF-1) (Yeo et al. 2017; Dongre and Weinberg 2019), or heat shock factor 1 (HSF1) / heat shock protein 90 (HSP90) (Dai 2018). Consequently, small-molecule inhibitors of HIF-1 (Gameiro et al. 2016; Zhang et al. 2015), HSF1 (Schilling et al. 2015), or HSP90 (Kudryavtsev et al. 2017) demonstrate a radiosensitizing effect on hypoxic tumors treated with high-LET radiation (Li et al. 2016; Lee et al. 2016). Additionally, RT combined with inhibitors targeting the PI3K/AKT/mTOR or HIF-1α pathways has been shown to enhance the radiosensitivity of endometrial cancer (Miyasaka et al. 2015; Song et al. 2024). This enhancement is achieved through the reducing autophagy, induction of apoptosis, inhibition of EMT, as well as suppression of NHEJ and HR repair mechanisms (Mardanshahi et al. 2021; Chang et al. 2014; Chang et al. 2013). Thus, combined approach may induce systemic antitumor immune responses, presenting a promising strategy for treating metastatic and difficult to treat cancer.

**Fig. 1:**
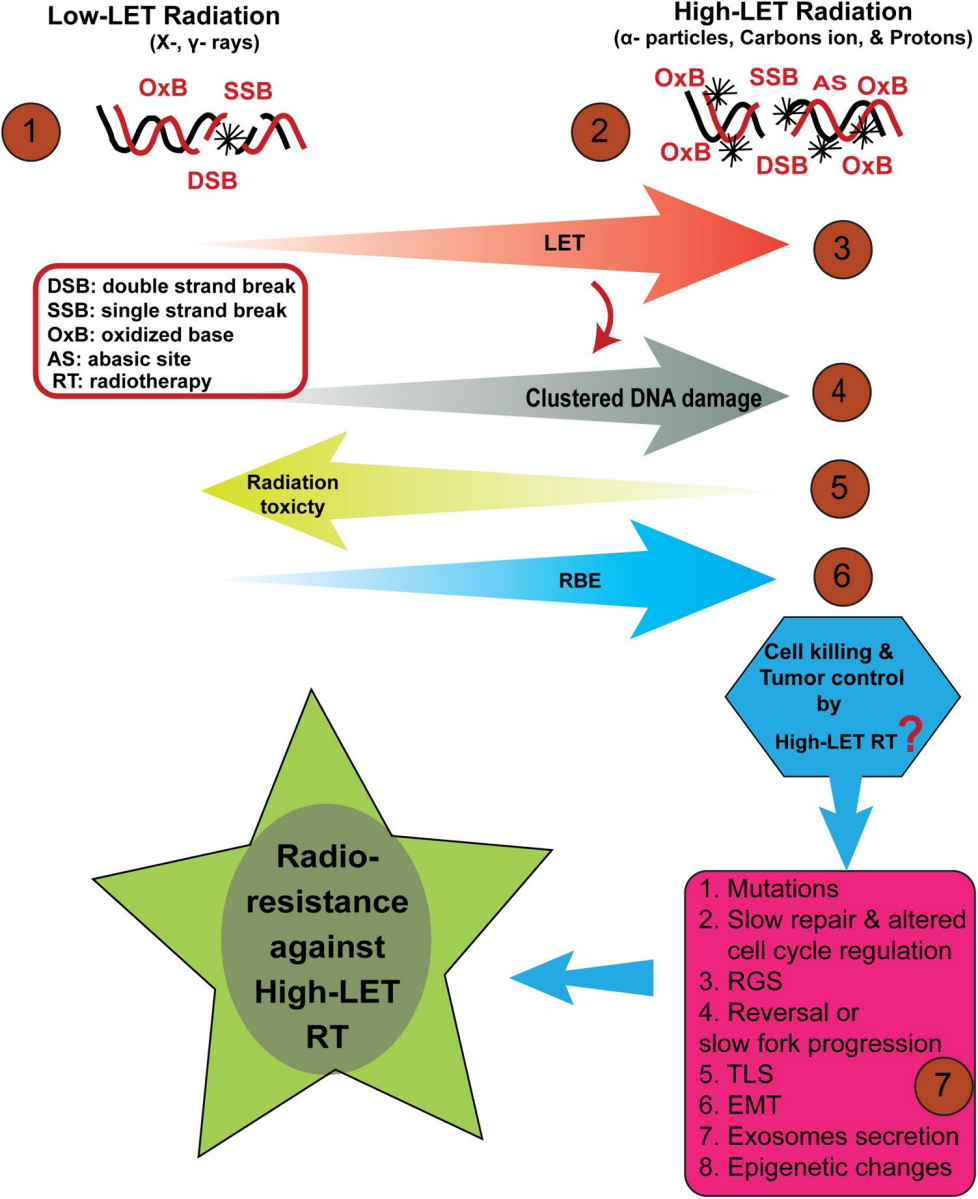
The biological effects and possible radiation resistance mechanisms against high-LET radiotherapy (RT). Cellular responses to both low and high-LET radiation offer valuable insights into the biological ramifications. Low-LET radiation (X-rays, γ-rays) causes sparsely distributed DNA damage (DSBs, SSBs, and oxidized bases) (1) in the genome, while high-LET radiation (α-particles, carbon ions, and protons) causes densely distributed clustered complex DNA damage (2). With increasing linear energy transfer (LET) (3), the density of clustered DNA damage increases markedly (4), while the reduction in radiation toxicity to healthy tissues is observed (5). However, the relative biological effectiveness (RBE) improves substantially (6). The cell killing and tumor control by high-LET RT are threatened by numerous molecular mechanisms (7). The possible mechanisms could be mutations incorporation, slow repair and altered cell cycle regulation, replication gap suppression (RGS), reversal or slow fork progression, translesion synthesis (TLS), epithelial‐mesenchymal transition (EMT) activation, release of exosomes, and epigenetic changes, which might confer radioresistance to tumor cells against high-LET RT, and may potentially, reduce the effectiveness of high-LET RT

Although high-LET RT has been shown to be beneficial over low-LET RT, apprehension and caution is warranted. Existing experimental evidence suggests that exposure to high-LET radiation is associated with an increased likelihood of cancer incidents (Luitel et al. 2018; Xie et al. 2020; Datta et al. 2013; Patel et al. 2020). Furthermore, high-LET RT has been shown to induce elevated levels of mutations, genomic instability, and chromosomal aberrations (Masumura et al. 2002; Yatagai et al. 2002), as well as elevated oxidative stress (Suman et al. 2018; Suman et al. 2013). Moreover, predictions from space exploration studies indicate a two-fold increase in excess relative risk (ERR) for male liver and female breast cancer due to high linear energy transfer (LET) radiation (Cucinotta 2022). High LET radiation also constitutes the primary source of uncertainty in galactic cosmic ray (GCR) studies (Cucinotta et al. 2020). The radioresistance observed against high-LET RT may be attributed to various damage bypass or avoidance mechanisms (Igarashi et al. 2023; Fu et al. 2024; Nayak, et al. 2020, 2021; Tonzi and Huang 2019; Saldanha, et al. 2023; Wu et al. 2023b; Li et al. 2019; He et al. 2022; Park et al. 2022; Venkadakrishnan et al. 2023; Nusawardhana et al. 2024), induction of autophagy (Chaachouay et al. 2011; Chen et al. 2015; Chen et al. 2011; Khan et al. 2020), RGS mechanisms (Cantor 2021; Cantor and Calvo 2017; Cong et al. 2024), replication fork reversal/slow movement (Zellweger et al. 2015; Bi 2015), EMT activation (Wu et al. 2023a), release of exosomes (Jokar, et al. 2022; Li et al. 2021a), and epigenetic changes (Kennedy et al. 2018; Perdyan et al. 2024; Tomsia et al. 2024) (Fig. 1). Consequently, the mutagenic potential of high-LET radiation appears to be transiently or persistently heightened. Concerns also arise regarding the delayed repair of clustered DNA damage, which could lead to mutations and the generation of DSBs from halted replication forks, particularly in rapidly dividing tumor cells. Additionally, clustered DSBs may undergo adaptive mutagenic repair, resulting in large and intricate deletions, as observed in cultured cells exposed to high-LET radiation (Michalettou, et al. 2021). Furthermore, genome loss contributes to the genomic instability of tumor cells, potentially leading to the death of the majority of tumor cells. However, rare surviving tumor cells may repopulate tumors with persistent growth due to adaptive mutability induced by high-LET radiation treatment, akin to bacterial cells. In bacterial cells, extensive generation of ssDNA gaps and unrepairable or challenging-to-repair DNA damage activate SOS responses, inducing adaptive mutagenesis (McKenzie et al. 2000; Fuchs 2016). Hence, it is crucial to carefully assess the potential of individual patient cancer cells to activate adaptive mutability and other molecular mechanisms responsible to bypass clustered DNA damage, thereby enhancing survival strategies. A comprehensive understanding of these bypass or survival strategies against clustered DNA damage is imperative on large cohorts studies over extended period for anticipating appropriate therapeutic outcomes from high-LET RT.

Furthermore, high-LET RT combined with various inhibitors approach may also pose risks to individual long-term survival due to increased genomic instability and genome restructuring. These consequences could lead to the resurgence of more aggressive tumor growth, as the tumor cells may develop adaptive resistance. Moreover, late toxicity, secondary cancers, and other adverse effects such as cataracts, fibrosis, vascular damage, immunological changes, endocrine disruptions, neurodegeneration could manifest over time (Ramaekers et al. 2011; Yang et al. 2022b; Nakashima et al. 2022; Hanna, et al. 2020; Shiba et al. 2022b; Chung et al. 2013). Additionally, while the increased energy deposition of high-LET radiation in nearby healthy tissue may not be significantly raised, the ramifications of this alteration remain undetermined (Blakely and Chang 2004). Unlike proton therapy, CIRT exhibits a fragmentation tail, wherein nuclear fragments contribute to the dose beyond the intended target area. This phenomenon introduces additional uncertainty, particularly in tissues situated distally from the target site (Malouff et al. 2020). While existing studies suggest that high-LET radiotherapy propensity to induce secondary malignancies is comparatively low compared to photon therapy, the presence of the fragmentation tail raises concerns. It is plausible that the development of secondary, radiation-induced cancers in adjacent healthy tissue may be facilitated by mutations in tumor suppressor genes (Chung et al. 2013; Dracham et al. 2018; Facoetti et al. 2019; Aherne and Murphy 2018; Kraus and Combs 2019; Yock and Caruso 2012; Mohamad et al. 2019).

Therefore, despite the increasing volume of related research and the proliferation of proton beam therapy (PBT) and/or CIRT centers, the clinical advantages of hadron-therapy treatments compared to conventional photon RT remain uncertain (Jefferson, et al. 2019). Ongoing clinical research may not resolve this uncertainty thus far due to a lack of consensus on appropriate study designs and insufficient collaboration among centers to develop comprehensive research protocols (Apisarnthanarax et al. 2018; Li et al. 2021b). Consequently, there has been a proliferation of small, inadequately designed, and poorly reported studies on this topic. These limitations may raise serious questions for the use of PBT and CIRT in experimental treatments, necessitating full disclosure of the risks and uncertainties to patients considering these therapies (Jefferson, et al. 2019; Goetz et al. 2019; Mishra et al. 2017; Fossati et al. 2018). Hence, it is imperative for clinicians and researchers to meticulously assess the therapeutic advantages of high-LET RT in a more comprehensive manner, considering the long-term suppression of tumor growth while minimizing detrimental health consequences. Researchers should focus on understanding cellular adaptive responses and strive to devise radiotherapy devices capable of inducing sparsely distributed clustered DNA damage instead of densely distributed DNA damage, thereby circumventing extreme cellular adaptive mechanisms such as RGS and the utilization of TLS for DNA damage bypass. Consequently, the notion of eradicating cancer cells without triggering their heightened adaptive mutability and facilitating DNA damage bypass should be prioritized.

## Conclusion

RT is a crucial therapeutic tool of cancer treatment, benefiting approximately half of cancer patients worldwide. High-LET radiation, notably in proton and hadron therapy, has gained global traction. However, a thorough comprehension of its biological mechanisms remains elusive. High-LET RT induces clustered DNA lesions through precision delivery of tumor radiation doses, potentially enhancing its tumor cell killing potential. Exploiting repair process vulnerabilities, such as BER and HR, alongside high-LET therapy, shows promise in augmenting tumor cell eradication. Nonetheless, caution is warranted due to cellular adaptive mutability supported by various molecular mechanisms (RGS, TLS, autophagy, EMT, release of exosomes, and epigenetic changes) which may lead to long-term toxicities and tumor relapse in the medium to longer term. Despite ongoing research, the clinical superiority of hadron therapy (high-LET) over conventional photon therapy (low-LET) remains inadequate. Addressing these uncertainties requires robust research protocols and transparent communication with patients. Therefore, a balanced evaluation of high-LET RT benefits and risks is imperative for informed clinical decisions.

## Data Availability

No datasets were generated or analysed during the current study.

## References

[CR1] Abbasi S et al (2021) The Ku complex: recent advances and emerging roles outside of non-homologous end-joining. Cell Mol Life Sci 78(10):4589–4613 33855626 10.1007/s00018-021-03801-1 PMC11071882

[CR2] Adewoye AB et al (2015) The genome-wide effects of ionizing radiation on mutation induction in the mammalian germline. Nat Commun 6:6684 25809527 10.1038/ncomms7684 PMC4389250

[CR3] Aherne NJ, Murphy BM (2018) Radiation-induced gliomas. Crit Rev Oncog 23(1–2):113–118 29953370 10.1615/CritRevOncog.2018025740

[CR4] Akagi T et al (2023) Formation of spread-out Bragg peak for helium-ion beam using microdosimetric kinetic model. Phys Med 109:102587 37087865 10.1016/j.ejmp.2023.102587

[CR5] Alemasova EE, Lavrik OI (2019) Poly(ADP-ribosyl)ation by PARP1: reaction mechanism and regulatory proteins. Nucleic Acids Res 47(8):3811–3827 30799503 10.1093/nar/gkz120 PMC6486540

[CR6] Allen C et al (2011) Heavy charged particle radiobiology: using enhanced biological effectiveness and improved beam focusing to advance cancer therapy. Mutat Res 711(1–2):150–157 21376738 10.1016/j.mrfmmm.2011.02.012 PMC3101308

[CR7] Anderson JA et al (2010) Participation of DNA-PKcs in DSB repair after exposure to high- and low-LET radiation. Radiat Res 174(2):195–205 20681786 10.1667/RR2071.1

[CR8] Antonovic L et al (2014) Clinical oxygen enhancement ratio of tumors in carbon ion radiotherapy: the influence of local oxygenation changes. J Radiat Res 55(5):902–911 24728013 10.1093/jrr/rru020 PMC4240637

[CR9] Apisarnthanarax S, Bowen SR, Combs SE (2018) Proton beam therapy and carbon ion radiotherapy for hepatocellular carcinoma. Semin Radiat Oncol 28(4):309–320 30309641 10.1016/j.semradonc.2018.06.008

[CR10] Arruebo M et al (2011) Assessment of the evolution of cancer treatment therapies. Cancers (basel) 3(3):3279–3330 24212956 10.3390/cancers3033279 PMC3759197

[CR11] Asaithamby A, Chen DJ (2011) Mechanism of cluster DNA damage repair in response to high-atomic number and energy particles radiation. Mutat Res 711(1–2):87–99 21126526 10.1016/j.mrfmmm.2010.11.002 PMC3318975

[CR12] Averbeck NB et al (2014) DNA end resection is needed for the repair of complex lesions in G1-phase human cells. Cell Cycle 13(16):2509–2516 25486192 10.4161/15384101.2015.941743 PMC4615131

[CR13] Aylon Y, Kupiec M (2003) The checkpoint protein Rad24 of *Saccharomyces cerevisiae* is involved in processing double-strand break ends and in recombination partner choice. Mol Cell Biol 23(18):6585–6596 12944484 10.1128/MCB.23.18.6585-6596.2003 PMC193701

[CR14] Bai G et al (2020) HLTF promotes fork reversal, limiting replication stress resistance and preventing multiple mechanisms of unrestrained DNA synthesis. Mol Cell 78(6):1237–12517 32442397 10.1016/j.molcel.2020.04.031 PMC7305998

[CR15] Barcellini A et al (2022) Ovarian cancer radiosensitivity: what have we understood so far? Life (basel) 13(1):6 36675955 10.3390/life13010006 PMC9861683

[CR16] Barnaba N, LaRocque JR (2021) Targeting cell cycle regulation via the G2-M checkpoint for synthetic lethality in melanoma. Cell Cycle 20(11):1041–1051 33966611 10.1080/15384101.2021.1922806 PMC8208119

[CR17] Baskar R et al (2012) Cancer and radiation therapy: current advances and future directions. Int J Med Sci 9(3):193–199 22408567 10.7150/ijms.3635 PMC3298009

[CR18] Bellon S et al (2009) Processing of thymine glycol in a clustered DNA damage site: mutagenic or cytotoxic. Nucleic Acids Res 37(13):4430–4440 19468043 10.1093/nar/gkp422 PMC2715253

[CR19] Bernal A et al (2024) The current therapeutic landscape for metastatic prostate cancer. Pharmaceuticals (basel) 17(3):351 38543137 10.3390/ph17030351 PMC10974045

[CR20] Betous R et al (2018) DNA replication stress triggers rapid DNA replication fork breakage by Artemis and XPF. PLoS Genet 14(7):e1007541 30059501 10.1371/journal.pgen.1007541 PMC6085069

[CR21] Bi X (2015) Mechanism of DNA damage tolerance. World J Biol Chem 6(3):48–56 26322163 10.4331/wjbc.v6.i3.48 PMC4549768

[CR22] Bi Y et al (2018) Radiosensitization by the PARP inhibitor olaparib in BRCA1-proficient and deficient high-grade serous ovarian carcinomas. Gynecol Oncol 150(3):534–544 30025822 10.1016/j.ygyno.2018.07.002

[CR23] Blakely EA, Chang PY (2004) Late effects from hadron therapy. Radiother Oncol 73(Suppl 2):S134–S140 15971329 10.1016/s0167-8140(04)80035-5

[CR24] Boguszewska K et al (2021) How (5’S) and (5’R) 5’,8-Cyclo-2’-deoxypurines affect base excision repair of clustered DNA damage in nuclear extracts of xrs5 cells? a biochemical study. Cells 10(4):725 33805115 10.3390/cells10040725 PMC8064110

[CR25] Borrego-Soto G, Ortiz-Lopez R, Rojas-Martinez A (2015) Ionizing radiation-induced DNA injury and damage detection in patients with breast cancer. Genet Mol Biol 38(4):420–432 26692152 10.1590/S1415-475738420150019 PMC4763322

[CR26] Bright SJ et al (2022) Targeted inhibition of DNA-PKcs, ATM, ATR, PARP, and Rad51 modulate response to X rays and protons. Radiat Res 198(4):336–346 35939823 10.1667/RADE-22-00040.1 PMC9648665

[CR27] Budworth H, Dianov GL (2003) Mode of inhibition of short-patch base excision repair by thymine glycol within clustered DNA lesions. J Biol Chem 278(11):9378–9381 12519757 10.1074/jbc.M212068200

[CR28] Bukowska B, Karwowski BT (2018) The clustered DNA lesions - types, pathways of repair and relevance to human health. Curr Med Chem 25(23):2722–2735 29484975 10.2174/0929867325666180226110502

[CR29] Busato F, Khouzai BE, Mognato M (2022) Biological mechanisms to reduce radioresistance and increase the efficacy of radiotherapy: state of the art. Int J Mol Sci 23(18):10211 36142122 10.3390/ijms231810211 PMC9499172

[CR30] Byrne S et al (2009) 5,6-Dihydrothymine impairs the base excision repair pathway of a closely opposed AP site or single-strand break. Radiat Res 172(5):537–549 19883221 10.1667/RR1830.1

[CR31] Caldecott KW (2019) XRCC1 protein form and function. DNA Repair (amst) 81:102664 31324530 10.1016/j.dnarep.2019.102664

[CR32] Cannan WJ, Pederson DS (2016) Mechanisms and consequences of double-strand DNA break formation in chromatin. J Cell Physiol 231(1):3–14 26040249 10.1002/jcp.25048 PMC4994891

[CR33] Cantor SB (2021) Revisiting the BRCA-pathway through the lens of replication gap suppression: “Gaps determine therapy response in BRCA Mutant cancer.” DNA Repair (amst) 107:103209 34419699 10.1016/j.dnarep.2021.103209 PMC9049047

[CR34] Cantor SB, Calvo JA (2017) Fork protection and therapy resistance in hereditary breast cancer. Cold Spring Harb Symp Quant Biol 82:339–348 29472318 10.1101/sqb.2017.82.034413 PMC6041132

[CR35] Cesaire M et al (2019) Sensitization of chondrosarcoma cells with PARP inhibitor and high-LET radiation. J Bone Oncol 17:100246 31312595 10.1016/j.jbo.2019.100246 PMC6609837

[CR36] Chaachouay H et al (2011) Autophagy contributes to resistance of tumor cells to ionizing radiation. Radiother Oncol 99(3):287–292 21722986 10.1016/j.radonc.2011.06.002

[CR37] Chang L et al (2013) Acquisition of epithelial-mesenchymal transition and cancer stem cell phenotypes is associated with activation of the PI3K/Akt/mTOR pathway in prostate cancer radioresistance. Cell Death Dis 4(10):e875 24157869 10.1038/cddis.2013.407 PMC3920940

[CR38] Chang L et al (2014) PI3K/Akt/mTOR pathway inhibitors enhance radiosensitivity in radioresistant prostate cancer cells through inducing apoptosis, reducing autophagy, suppressing NHEJ and HR repair pathways. Cell Death Dis 5(10):e1437 25275598 10.1038/cddis.2014.415 PMC4237243

[CR39] Chatterjee N, Walker GC (2017) Mechanisms of DNA damage, repair, and mutagenesis. Environ Mol Mutagen 58(5):235–263 28485537 10.1002/em.22087 PMC5474181

[CR40] Chen YS et al (2011) Autophagy inhibition contributes to radiation sensitization of esophageal squamous carcinoma cells. Dis Esophagus 24(6):437–443 21166739 10.1111/j.1442-2050.2010.01156.x

[CR41] Chen Y et al (2015) Combining radiation with autophagy inhibition enhances suppression of tumor growth and angiogenesis in esophageal cancer. Mol Med Rep 12(2):1645–1652 25891159 10.3892/mmr.2015.3623 PMC4464360

[CR42] Chen S et al (2021) Human MUS81: a fence-sitter in cancer. Front Cell Dev Biol 9:657305 33791310 10.3389/fcell.2021.657305 PMC8005573

[CR43] Chung CS et al (2013) Incidence of second malignancies among patients treated with proton versus photon radiation. Int J Radiat Oncol Biol Phys 87(1):46–52 23778197 10.1016/j.ijrobp.2013.04.030

[CR44] Cong K et al (2021) Replication gaps are a key determinant of PARP inhibitor synthetic lethality with BRCA deficiency. Mol Cell 81(15):3128-3144 e7 34216544 10.1016/j.molcel.2021.06.011 PMC9089372

[CR45] Cong K et al (2024) FANCJ promotes PARP1 activity during DNA replication that is essential in BRCA1 deficient cells. Nat Commun 15(1):2599 38521768 10.1038/s41467-024-46824-5 PMC10960859

[CR46] Cortes-Ciriano I et al (2023) Author correction: comprehensive analysis of chromothripsis in 2,658 human cancers using whole-genome sequencing. Nat Genet 55(6):1076 36944733 10.1038/s41588-023-01315-z PMC10260391

[CR47] Crisafulli G, Siravegna G (2023) Editorial: the impact of genetics on CRC therapy: from adaptive mutability to drug resistance. Front Oncol 13:1260158 37614500 10.3389/fonc.2023.1260158 PMC10443090

[CR48] Cuadrado M et al (2006) ATM regulates ATR chromatin loading in response to DNA double-strand breaks. J Exp Med 203(2):297–303 16461339 10.1084/jem.20051923 PMC2118201

[CR49] Cucinotta FA (2022) Flying without a net: space radiation cancer risk predictions without a gamma-ray basis. Int J Mol Sci 23(8):4324 35457139 10.3390/ijms23084324 PMC9029417

[CR50] Cucinotta FA et al (2020) Benchmarking risk predictions and uncertainties in the NSCR model of GCR cancer risks with revised low let risk coefficients. Life Sci Space Res (amst) 27:64–73 34756232 10.1016/j.lssr.2020.07.008

[CR51] Cunniffe S et al (2014a) Increased mutability and decreased repairability of a three-lesion clustered DNA-damaged site comprised of an AP site and bi-stranded 8-oxoG lesions. Int J Radiat Biol 90(6):468–479 24597750 10.3109/09553002.2014.899449 PMC4059193

[CR52] Cunniffe S et al (2014b) Reduced repair capacity of a DNA clustered damage site comprised of 8-oxo-7,8-dihydro-2’-deoxyguanosine and 2-deoxyribonolactone results in an increased mutagenic potential of these lesions. Mutat Res 762:32–39 24631220 10.1016/j.mrfmmm.2014.02.005 PMC3990186

[CR53] Dagogo-Jack I, Shaw AT (2018) Tumour heterogeneity and resistance to cancer therapies. Nat Rev Clin Oncol 15(2):81–94 29115304 10.1038/nrclinonc.2017.166

[CR54] Dai C (2018) The heat-shock, or HSF1-mediated proteotoxic stress, response in cancer: from proteomic stability to oncogenesis. Philos Trans R Soc Lond B Biol Sci 373:1738 10.1098/rstb.2016.0525 PMC5717525 29203710

[CR55] Danforth JM, Provencher L, Goodarzi AA (2022) Chromatin and the cellular response to particle radiation-induced oxidative and clustered DNA damage. Front Cell Dev Biol 10:910440 35912116 10.3389/fcell.2022.910440 PMC9326100

[CR56] Darafsheh A et al (2021) spread-out Bragg peak proton FLASH irradiation using a clinical synchrocyclotron: PROOF of concept and ion chamber characterization. Med Phys 48(8):4472–4484 34077590 10.1002/mp.15021

[CR57] Datta K, Neumann RD, Winters TA (2005) Characterization of complex apurinic/apyrimidinic-site clustering associated with an authentic site-specific radiation-induced DNA double-strand break. Proc Natl Acad Sci U S A 102(30):10569–10574 16024726 10.1073/pnas.0503975102 PMC1180784

[CR58] Datta K et al (2013) Heavy ion radiation exposure triggered higher intestinal tumor frequency and greater beta-catenin activation than gamma radiation in APC(Min/+) mice. PLoS ONE 8(3):e59295 23555653 10.1371/journal.pone.0059295 PMC3605451

[CR59] de Kruijff RM, Wolterbeek HT, Denkova AG (2015) A critical review of alpha radionuclide therapy-how to deal with recoiling daughters? Pharmaceuticals (basel) 8(2):321–336 26066613 10.3390/ph8020321 PMC4491664

[CR60] Debela DT et al (2021) New approaches and procedures for cancer treatment: Current perspectives. SAGE Open Med 9:20503121211034370 34408877 10.1177/20503121211034366 PMC8366192

[CR61] Deng S et al (2022) Targeting the DNA damage response and DNA repair pathways to enhance radiosensitivity in colorectal cancer. Cancers (basel) 14(19):4874 36230796 10.3390/cancers14194874 PMC9561988

[CR62] Deorukhkar A, Krishnan S (2010) Targeting inflammatory pathways for tumor radiosensitization. Biochem Pharmacol 80(12):1904–1914 20599771 10.1016/j.bcp.2010.06.039 PMC3090731

[CR63] Dietlein F, Reinhardt HC (2014) Molecular pathways: exploiting tumor-specific molecular defects in DNA repair pathways for precision cancer therapy. Clin Cancer Res 20(23):5882–5887 25451105 10.1158/1078-0432.CCR-14-1165

[CR64] Dietlein F, Thelen L, Reinhardt HC (2014) Cancer-specific defects in DNA repair pathways as targets for personalized therapeutic approaches. Trends Genet 30(8):326–339 25017190 10.1016/j.tig.2014.06.003

[CR65] Dongre A, Weinberg RA (2019) New insights into the mechanisms of epithelial-mesenchymal transition and implications for cancer. Nat Rev Mol Cell Biol 20(2):69–84 30459476 10.1038/s41580-018-0080-4

[CR66] Dracham CB, Shankar A, Madan R (2018) Radiation induced secondary malignancies: a review article. Radiat Oncol J 36(2):85–94 29983028 10.3857/roj.2018.00290 PMC6074073

[CR67] Du TQ et al (2022) Does particle radiation have superior radiobiological advantages for prostate cancer cells? a systematic review of in vitro studies. Eur J Med Res 27(1):306 36572945 10.1186/s40001-022-00942-2 PMC9793637

[CR68] Eccles LJ, O’Neill P, Lomax ME (2011) Delayed repair of radiation induced clustered DNA damage: friend or foe? Mutat Res 711(1–2):134–141 21130102 10.1016/j.mrfmmm.2010.11.003 PMC3112496

[CR69] Eckelmann BJ et al (2020) XRCC1 promotes replication restart, nascent fork degradation and mutagenic DNA repair in BRCA2-deficient cells. NAR Cancer 2(3):zcaa013 32776008 10.1093/narcan/zcaa013 PMC7397735

[CR70] Eot-Houllier G et al (2005) Processing of a complex multiply damaged DNA site by human cell extracts and purified repair proteins. Nucleic Acids Res 33(1):260–271 15647508 10.1093/nar/gki165 PMC546153

[CR71] Facoetti A et al (2019) The role of particle therapy in the risk of radio-induced second tumors: a review of the literature. Anticancer Res 39(9):4613–4617 31519558 10.21873/anticanres.13641

[CR72] Ferlay J et al (2021) Cancer statistics for the year 2020: an overview. Int J Cancer 149:778–779 10.1002/ijc.33588 33818764

[CR73] Fossati P et al (2018) Radiobiological issues in prospective carbon ion therapy trials. Med Phys 45(11):e1096–e1110 30421806 10.1002/mp.12506

[CR74] Fu X et al (2024) Mechanism of PARP inhibitor resistance and potential overcoming strategies. Genes Dis 11(1):306–320 37588193 10.1016/j.gendis.2023.02.014 PMC10425807

[CR75] Fuchs RP (2016) Tolerance of lesions in *E coli*: chronological competition between translesion synthesis and damage avoidance. DNA Repair (amst) 44:51–58 27321147 10.1016/j.dnarep.2016.05.006

[CR76] Fujisawa H et al (2015) VE-821, an ATR inhibitor, causes radiosensitization in human tumor cells irradiated with high LET radiation. Radiat Oncol 10:175 26286029 10.1186/s13014-015-0464-y PMC4554350

[CR77] Gameiro SR et al (2016) Tumor cells surviving exposure to proton or photon radiation share a common immunogenic modulation signature, rendering them more sensitive to T Cell-mediated killing. Int J Radiat Oncol Biol Phys 95(1):120–130 27084634 10.1016/j.ijrobp.2016.02.022 PMC4834148

[CR78] Georgakilas AG et al (2004) Processing of bistranded abasic DNA clusters in gamma-irradiated human hematopoietic cells. Nucleic Acids Res 32(18):5609–5620 15494449 10.1093/nar/gkh871 PMC524283

[CR79] Georgakilas AG, O’Neill P, Stewart RD (2013) Induction and repair of clustered DNA lesions: what do we know so far? Radiat Res 180(1):100–109 23682596 10.1667/RR3041.1

[CR80] Georgoulis A et al (2017) Genome instability and gammaH2AX. Int J Mol Sci 18(9):1979 28914798 10.3390/ijms18091979 PMC5618628

[CR81] Gerelchuluun A et al (2015) The major DNA repair pathway after both proton and carbon-ion radiation is NHEJ, but the HR pathway is more relevant in carbon ions. Radiat Res 183(3):345–356 25738894 10.1667/RR13904.1 PMC5684887

[CR82] Glanzer JG et al (2014) RPA inhibition increases replication stress and suppresses tumor growth. Cancer Res 74(18):5165–5172 25070753 10.1158/0008-5472.CAN-14-0306 PMC4201622

[CR83] Goetz G et al (2019) Health technology assessment of carbon-ion beam radiotherapy: a systematic review of clinical effectiveness and safety for 54 oncological indications in 12 tumour regions. Anticancer Res 39(4):1635–1650 30952702 10.21873/anticanres.13269

[CR84] Goodarzi AA, Block WD, Lees-Miller SP (2003) The role of ATM and ATR in DNA damage-induced cell cycle control. Prog Cell Cycle Res 5:393–411 14593734

[CR85] Goodhead DT, Nikjoo H (1989) Track structure analysis of ultrasoft X-rays compared to high- and low-LET radiations. Int J Radiat Biol 55(4):513–529 2564863 10.1080/09553008914550571

[CR86] Hada M, Georgakilas AG (2008) Formation of clustered DNA damage after high-LET irradiation: a review. J Radiat Res 49(3):203–210 18413977 10.1269/jrr.07123

[CR87] Hanna C et al (2020) Treatment of newly diagnosed glioblastoma in the elderly: a network meta-analysis. Cochrane Database Syst Rev 3(3):CD013261 32202316 10.1002/14651858.CD013261.pub2 PMC7086476

[CR88] Hanscom T, McVey M (2020) Regulation of error-prone DNA double-strand break repair and its impact on genome evolution. Cells 9(7):1657 32660124 10.3390/cells9071657 PMC7407515

[CR89] Hanssen-Bauer A et al (2012) X-ray repair cross complementing protein 1 in base excision repair. Int J Mol Sci 13(12):17210–17229 23247283 10.3390/ijms131217210 PMC3546746

[CR90] Harper JV, Anderson JA, O’Neill P (2010) Radiation induced DNA DSBs: Contribution from stalled replication forks? DNA Repair (amst) 9(8):907–913 20634148 10.1016/j.dnarep.2010.06.002

[CR91] Harrison L, Hatahet Z, Wallace SS (1999) In vitro repair of synthetic ionizing radiation-induced multiply damaged DNA sites. J Mol Biol 290(3):667–684 10395822 10.1006/jmbi.1999.2892

[CR92] Hayashi K et al (2019) Feasibility of Re-irradiation using carbon ions for recurrent head and neck malignancies after carbon-ion radiotherapy. Radiother Oncol 136:148–153 31015117 10.1016/j.radonc.2019.04.007

[CR93] Hays E et al (2020) The SWI/SNF ATPase BRG1 stimulates DNA end resection and homologous recombination by reducing nucleosome density at DNA double strand breaks and by promoting the recruitment of the CtIP nuclease. Cell Cycle 19(22):3096–3114 33044911 10.1080/15384101.2020.1831256 PMC7714457

[CR94] He J et al (2022) The roles of EXO1 and RPA1 polymorphisms in prognosis of lung cancer patients treated with platinum-based chemotherapy. Dis Markers 2022:3306189 36277983 10.1155/2022/3306189 PMC9584701

[CR95] Higuchi T et al (2015) CTLA-4 blockade synergizes therapeutically with PARP inhibition in BRCA1-deficient ovarian cancer. Cancer Immunol Res 3(11):1257–1268 26138335 10.1158/2326-6066.CIR-15-0044 PMC4984269

[CR96] Hirai T et al (2012) Radiosensitization effect of poly(ADP-ribose) polymerase inhibition in cells exposed to low and high liner energy transfer radiation. Cancer Sci 103(6):1045–1050 22404155 10.1111/j.1349-7006.2012.02268.x PMC7685087

[CR97] Hsu CW et al (2022) A combinatorial system to examine the enzymatic repair of multiply damaged DNA substrates. Nucleic Acids Res 50(13):7406–7419 35776119 10.1093/nar/gkac530 PMC9303388

[CR98] Huang RX, Zhou PK (2020) DNA damage response signaling pathways and targets for radiotherapy sensitization in cancer. Signal Transduct Target Ther 5(1):60 32355263 10.1038/s41392-020-0150-x PMC7192953

[CR99] Huang L et al (2023) High-throughput sequencing reveals Jatrorrhizine inhibits colorectal cancer growth by ferroptosis-related genes. BMC Med Genomics 16(1):217 37710311 10.1186/s12920-023-01619-3 PMC10500743

[CR100] Igarashi T et al (2023) An ATR-PrimPol pathway confers tolerance to oncogenic KRAS-induced and heterochromatin-associated replication stress. Nat Commun 14(1):4991 37591859 10.1038/s41467-023-40578-2 PMC10435487

[CR101] Jackson SP (2002) Sensing and repairing DNA double-strand breaks. Carcinogenesis 23(5):687–696 12016139 10.1093/carcin/23.5.687

[CR102] Jefferson T et al (2019) Hadrontherapy for cancer. an overview of HTA reports and ongoing studies. Recenti Prog Med 110(12):566–586 31909760 10.1701/3278.32516

[CR103] Jiang Y, Wang Y, Wang Y (2009) In vitro replication and repair studies of tandem lesions containing neighboring thymidine glycol and 8-oxo-7,8-dihydro-2’-deoxyguanosine. Chem Res Toxicol 22(3):574–583 19193190 10.1021/tx8003449 PMC2765499

[CR104] Jokar S et al (2022) The footprint of exosomes in the radiation-induced bystander effects. Bioengineering (basel) 9(6):243 35735486 10.3390/bioengineering9060243 PMC9220715

[CR105] Kabakov AE, Yakimova AO (2021) Hypoxia-induced cancer cell responses driving radioresistance of hypoxic tumors: approaches to targeting and radiosensitizing. Cancers (basel) 13(5):1102 33806538 10.3390/cancers13051102 PMC7961562

[CR106] Kamada T et al (2015) Carbon ion radiotherapy in Japan: an assessment of 20 years of clinical experience. Lancet Oncol 16(2):e93–e100 25638685 10.1016/S1470-2045(14)70412-7

[CR107] Kamaletdinova T, Fanaei-Kahrani Z, Wang ZQ (2019) The enigmatic function of PARP1: from PARYLATION activity to PAR readers. Cells 8(12):1625 31842403 10.3390/cells8121625 PMC6953017

[CR108] Karwowski BT (2019) The influence of (5’R)- and (5’S)-5’,8-Cyclo-2’-deoxyadenosine on UDG and hAPE1 activity. tandem lesions are the base excision repair system’s nightmare. Cells 8(11):1303 31652769 10.3390/cells8111303 PMC6912673

[CR109] Karwowski BT (2021) (5’S) 5’,8-Cyclo-2’-deoxyadenosine cannot Stop BER. clustered DNA lesion studies. Int J Mol Sci 22(11):5934 34072994 10.3390/ijms22115934 PMC8199134

[CR110] Kazmierczak-Baranska J et al (2021) Effects of 5’,8’-Cyclo-2’-deoxypurines on the base excision repair of clustered DNA lesions in nuclear extracts of the XPC cell line. Cells 10(11):3254 34831476 10.3390/cells10113254 PMC8618216

[CR111] Kennedy EM et al (2018) Galactic cosmic radiation induces persistent epigenome alterations relevant to human lung cancer. Sci Rep 8(1):6709 29712937 10.1038/s41598-018-24755-8 PMC5928241

[CR112] Khan T et al (2020) Autophagy modulators for the treatment of oral and esophageal squamous cell carcinomas. Med Res Rev 40(3):1002–1060 31742748 10.1002/med.21646

[CR113] Kiseleva V et al (2022) Particle therapy: clinical applications and biological effects. Life (basel) 12(12):2071 36556436 10.3390/life12122071 PMC9785772

[CR114] Kong X et al (2021) Relationship between p53 status and the bioeffect of ionizing radiation. Oncol Lett 22(3):661 34386083 10.3892/ol.2021.12922 PMC8299044

[CR115] Kozmin SG et al (2021) Dissecting highly mutagenic processing of complex clustered DNA damage in yeast *Saccharomyces cerevisiae*. Cells 10(9):2309 34571958 10.3390/cells10092309 PMC8471780

[CR116] Kraus KM, Combs SE (2019) The risk of subsequent primary cancer after radiotherapy of localised prostate cancer with carbon ions may be reduced compared to treatment with photons. Strahlenther Onkol 195(11):1033–1035 31420708 10.1007/s00066-019-01510-0

[CR117] Kudryavtsev VA et al (2017) Induction of Hsp70 in tumor cells treated with inhibitors of the Hsp90 activity: a predictive marker and promising target for radiosensitization. PLoS ONE 12(3):e0173640 28291803 10.1371/journal.pone.0173640 PMC5349677

[CR118] Kumar K et al (2023) High-LET-radiation-induced persistent DNA damage response signaling and gastrointestinal cancer development. Curr Oncol 30(6):5497–5514 37366899 10.3390/curroncol30060416 PMC10297158

[CR119] Lee Y et al (2016) The purine scaffold Hsp90 inhibitor PU-H71 sensitizes cancer cells to heavy ion radiation by inhibiting DNA repair by homologous recombination and non-homologous end joining. Radiother Oncol 121(1):162–168 27666928 10.1016/j.radonc.2016.08.029 PMC5110220

[CR120] Li F, Sethi G (2010) Targeting transcription factor NF-kappaB to overcome chemoresistance and radioresistance in cancer therapy. Biochim Biophys Acta 1805(2):167–180 20079806 10.1016/j.bbcan.2010.01.002

[CR121] Li HK et al (2016) PU-H71, a novel Hsp90 inhibitor, as a potential cancer-specific sensitizer to carbon-ion beam therapy. J Radiat Res 57(5):572–575 27242340 10.1093/jrr/rrw054 PMC5045081

[CR122] Li S et al (2019) Genetic variants in RPA1 associated with the response to oxaliplatin-based chemotherapy in colorectal cancer. J Gastroenterol 54(11):939–949 30923916 10.1007/s00535-019-01571-z

[CR123] Li F et al (2021) Shift in G(1)-checkpoint from ATM-alone to a cooperative ATM Plus ATR regulation with increasing dose of radiation. Cells 11(1):63 35011623 10.3390/cells11010063 PMC8750242

[CR124] Li Z et al (2021a) Characterization of exosome release and extracellular vesicle-associated miRNAs for human bronchial epithelial cells irradiated with high charge and energy ions. Life Sci Space Res (amst) 28:11–17 33612174 10.1016/j.lssr.2020.11.001

[CR125] Li M et al (2021b) Clinical efficacy and safety of proton and carbon ion radiotherapy for prostate cancer: a systematic review and meta-analysis. Front Oncol 11:709530 34712607 10.3389/fonc.2021.709530 PMC8547329

[CR126] Lin YF et al (2018) The relative biological effect of spread-out bragg peak protons in sensitive and resistant tumor cells. Int J Part Ther 4(3):33–39 30283809 10.14338/IJPT-17-00025.1 PMC6167011

[CR127] Liu X et al (2018) Genistein sensitizes glioblastoma cells to carbon ions via inhibiting DNA-PKcs phosphorylation and subsequently repressing NHEJ and delaying HR repair pathways. Radiother Oncol 129(1):84–94 29685705 10.1016/j.radonc.2018.04.005

[CR128] Liu YP et al (2021) Molecular mechanisms of chemo- and radiotherapy resistance and the potential implications for cancer treatment. MedComm 2(3):315–340 34766149 10.1002/mco2.55 PMC8554658

[CR129] Liu J et al (2022) Wnt/beta-catenin signalling: function, biological mechanisms, and therapeutic opportunities. Signal Transduct Target Ther 7(1):3 34980884 10.1038/s41392-021-00762-6 PMC8724284

[CR130] Lobrich M, Jeggo PA (2005) Harmonising the response to DSBs: a new string in the ATM bow. DNA Repair (amst) 4(7):749–759 15978533 10.1016/j.dnarep.2004.12.008

[CR131] Lomax ME, Cunniffe S, O’Neill P (2004a) 8-OxoG retards the activity of the ligase III/XRCC1 complex during the repair of a single-strand break, when present within a clustered DNA damage site. DNA Repair (amst) 3(3):289–299 15177044 10.1016/j.dnarep.2003.11.006

[CR132] Lomax ME, Cunniffe S, O’Neill P (2004b) Efficiency of repair of an abasic site within DNA clustered damage sites by mammalian cell nuclear extracts. Biochemistry 43(34):11017–11026 15323560 10.1021/bi049560r

[CR133] Lorat Y et al (2015) Nanoscale analysis of clustered DNA damage after high-LET irradiation by quantitative electron microscopy–the heavy burden to repair. DNA Repair (amst) 28:93–106 25659339 10.1016/j.dnarep.2015.01.007

[CR134] Lorat Y et al (2016) Clustered double-strand breaks in heterochromatin perturb DNA repair after high linear energy transfer irradiation. Radiother Oncol 121(1):154–161 27637859 10.1016/j.radonc.2016.08.028

[CR135] Luitel K et al (2018) Proton radiation-induced cancer progression. Life Sci Space Res (amst) 19:31–42 30482279 10.1016/j.lssr.2018.08.002

[CR136] Ma H et al (2015) Combining carbon ion irradiation and non-homologous end-joining repair inhibitor NU7026 efficiently kills cancer cells. Radiat Oncol 10:225 26553138 10.1186/s13014-015-0536-z PMC4638098

[CR137] Malouff TD et al (2020) Carbon ion therapy: a modern review of an emerging technology. Front Oncol 10:82 32117737 10.3389/fonc.2020.00082 PMC7010911

[CR138] Malyarchuk S, Harrison L (2005) DNA repair of clustered uracils in HeLa cells. J Mol Biol 345(4):731–743 15588822 10.1016/j.jmb.2004.10.079

[CR139] Malyarchuk S et al (2003) The mutation frequency of 8-oxo-7,8-dihydroguanine (8-oxodG) situated in a multiply damaged site: comparison of a single and two closely opposed 8-oxodG in Escherichia coli. DNA Repair (amst) 2(6):695–705 12767348 10.1016/s1568-7864(03)00040-5

[CR140] Malyarchuk S et al (2004) Two clustered 8-oxo-7,8-dihydroguanine (8-oxodG) lesions increase the point mutation frequency of 8-oxodG, but do not result in double strand breaks or deletions in Escherichia coli. Nucleic Acids Res 32(19):5721–5731 15509868 10.1093/nar/gkh911 PMC528796

[CR141] Malyarchuk S, Castore R, Harrison L (2008) DNA repair of clustered lesions in mammalian cells: involvement of non-homologous end-joining. Nucleic Acids Res 36(15):4872–4882 18653525 10.1093/nar/gkn450 PMC2528178

[CR142] Malyarchuk S, Castore R, Harrison L (2009) Apex1 can cleave complex clustered DNA lesions in cells. DNA Repair (amst) 8(12):1343–1354 19800300 10.1016/j.dnarep.2009.08.008 PMC2801153

[CR143] Mardanshahi A et al (2021) The PI3K/AKT/mTOR signaling pathway inhibitors enhance radiosensitivity in cancer cell lines. Mol Biol Rep 48(8):1–14 34357550 10.1007/s11033-021-06607-3

[CR144] Marechal A, Zou L (2013) DNA damage sensing by the ATM and ATR kinases. Cold Spring Harb Perspect Biol 5(9):a012716 24003211 10.1101/cshperspect.a012716 PMC3753707

[CR145] Marini V et al (2023) MUS81 cleaves TOP1-derived lesions and other DNA-protein cross-links. BMC Biol 21(1):110 37194054 10.1186/s12915-023-01614-1 PMC10189953

[CR146] Masumura K et al (2002) Heavy-ion-induced mutations in the gpt delta transgenic mouse: comparison of mutation spectra induced by heavy-ion, X-ray, and gamma-ray radiation. Environ Mol Mutagen 40(3):207–215 12355555 10.1002/em.10108

[CR147] McKenzie GJ et al (2000) The SOS response regulates adaptive mutation. Proc Natl Acad Sci U S A 97(12):6646–6651 10829077 10.1073/pnas.120161797 PMC18688

[CR148] Michalettou TD et al (2021) A Meta-analysis of the effects of high-LET ionizing radiations in human gene expression. Life (basel) 11(2):115 33546472 10.3390/life11020115 PMC7913660

[CR149] Mishra MV et al (2017) Establishing evidence-based indications for proton therapy: an overview of current clinical trials. Int J Radiat Oncol Biol Phys 97(2):228–235 28068231 10.1016/j.ijrobp.2016.10.045

[CR150] Miyasaka A et al (2015) PI3K/mTOR pathway inhibition overcomes radioresistance via suppression of the HIF1-alpha/VEGF pathway in endometrial cancer. Gynecol Oncol 138(1):174–180 25913131 10.1016/j.ygyno.2015.04.015

[CR151] Mladenov E et al (2019a) Radiation-dose-dependent functional synergisms between ATM, ATR and DNA-PKcs in checkpoint control and resection in G(2)-phase. Sci Rep 9(1):8255 31164689 10.1038/s41598-019-44771-6 PMC6547644

[CR152] Mladenov E et al (2019b) DNA-PKcs and ATM epistatically suppress DNA end resection and hyperactivation of ATR-dependent G(2)-checkpoint in S-phase irradiated cells. Sci Rep 9(1):14597 31601897 10.1038/s41598-019-51071-6 PMC6787047

[CR153] Mladenova V et al (2022) DNA damage clustering after ionizing radiation and consequences in the processing of chromatin breaks. Molecules 27(5):1540 35268641 10.3390/molecules27051540 PMC8911773

[CR154] Mladenova V et al (2022) The high toxicity of DSB-clusters modelling high-LET-DNA damage derives from inhibition of c-NHEJ and promotion of alt-EJ and SSA despite increases in HR. Front Cell Dev Biol 10:1016951 36263011 10.3389/fcell.2022.1016951 PMC9574094

[CR155] Mohamad O et al (2017) Carbon ion radiotherapy: a review of clinical experiences and preclinical research, with an emphasis on DNA damage/repair. Cancers (basel) 9(6):66 28598362 10.3390/cancers9060066 PMC5483885

[CR156] Mohamad O et al (2019) Risk of subsequent primary cancers after carbon ion radiotherapy, photon radiotherapy, or surgery for localised prostate cancer: a propensity score-weighted, retrospective, cohort study. Lancet Oncol 20(5):674–685 30885458 10.1016/S1470-2045(18)30931-8

[CR157] Mohammadian Gol T, Rodemann HP, Dittmann K (2019) Depletion of Akt1 and Akt2 impairs the repair of radiation-induced DNA double strand breaks via homologous recombination. Int J Mol Sci 20(24):6316 31847370 10.3390/ijms20246316 PMC6941063

[CR158] Mondini M et al (2020) Radiotherapy-immunotherapy combinations - perspectives and challenges. Mol Oncol 14(7):1529–1537 32112478 10.1002/1878-0261.12658 PMC7332212

[CR159] Moo TA et al (2018) Overview of breast cancer therapy. PET Clin 13(3):339–354 30100074 10.1016/j.cpet.2018.02.006 PMC6092031

[CR160] Morales-Ramirez P et al (2022) Induction and assessment of persistent radioresistance in murine leukocytes in vivo. Biochem Biophys Rep 31:101296 35707716 10.1016/j.bbrep.2022.101296 PMC9189778

[CR161] Mourgues S, Lomax ME, O’Neill P (2007) Base excision repair processing of abasic site/single-strand break lesions within clustered damage sites associated with XRCC1 deficiency. Nucleic Acids Res 35(22):7676–7687 17982170 10.1093/nar/gkm947 PMC2190709

[CR162] Mozaffari NL, Pagliarulo F, Sartori AA (2021) Human CtIP: A “double agent” in DNA repair and tumorigenesis. Semin Cell Dev Biol 113:47–56 32950401 10.1016/j.semcdb.2020.09.001

[CR163] Musha A et al (2022) Carbon-ion radiotherapy for inoperable head and neck bone and soft-tissue sarcoma: prospective observational study. Anticancer Res 42(3):1439–1446 35220237 10.21873/anticanres.15614

[CR164] Nakashima H, Takatsu T, Imai R (2022) Radiation-induced osteosarcoma in the pubic bone after proton radiotherapy for prostate cancer: a case report. J Rural Med 17(2):94–100 35432636 10.2185/jrm.2021-047 PMC8984621

[CR165] Naldiga S et al (2020) Mutagenic effects of a 2-deoxyribonolactone-thymine glycol Tandem DNA lesion in human cells. Biochemistry 59(4):417–424 31860280 10.1021/acs.biochem.9b01058 PMC7003646

[CR166] Nayak S et al (2020) Inhibition of the translesion synthesis polymerase REV1 exploits replication gaps as a cancer vulnerability. Sci Adv 6(24):eaaz7808 32577513 10.1126/sciadv.aaz7808 PMC7286678

[CR167] Nayak S, Calvo JA, Cantor SB (2021) Targeting translesion synthesis (TLS) to expose replication gaps, a unique cancer vulnerability. Expert Opin Ther Targets 25(1):27–36 33416413 10.1080/14728222.2021.1864321 PMC7837368

[CR168] Nickoloff JA, Sharma N, Taylor L (2020) Clustered DNA double-strand breaks: biological effects and relevance to cancer radiotherapy. Genes (basel) 11(1):99 31952359 10.3390/genes11010099 PMC7017136

[CR169] Nickoloff JA et al (2022) Nucleases and Co-factors in DNA replication stress responses. DNA (basel) 2(1):68–85 36203968 10.3390/dna2010006 PMC9534323

[CR170] Nikitaki Z et al (2016) Measurement of complex DNA damage induction and repair in human cellular systems after exposure to ionizing radiations of varying linear energy transfer (LET). Free Radic Res 50(sup1):S64–S78 27593437 10.1080/10715762.2016.1232484

[CR171] Nikjoo H et al (1998) Track structure in radiation biology: theory and applications. Int J Radiat Biol 73(4):355–364 9587072 10.1080/095530098142176

[CR172] Niu Y et al (2013) XRCC1 deficiency increased the DNA damage induced by gamma-ray in HepG2 cell: involvement of DSB repair and cell cycle arrest. Environ Toxicol Pharmacol 36(2):311–319 23708312 10.1016/j.etap.2013.04.009

[CR173] Noordermeer SM et al (2018) The shieldin complex mediates 53BP1-dependent DNA repair. Nature 560(7716):117–121 30022168 10.1038/s41586-018-0340-7 PMC6141009

[CR174] Nusawardhana A et al (2024) USP1-dependent nucleolytic expansion of PRIMPOL-generated nascent DNA strand discontinuities during replication stress. Nucleic Acids Res 52(5):2340–2354 38180818 10.1093/nar/gkad1237 PMC10954467

[CR175] Oden J, Eriksson K, Toma-Dasu I (2017) Inclusion of a variable RBE into proton and photon plan comparison for various fractionation schedules in prostate radiation therapy. Med Phys 44(3):810–822 28107554 10.1002/mp.12117

[CR176] Okada T et al (2010) Carbon ion radiotherapy: clinical experiences at National Institute of Radiological Science (NIRS). J Radiat Res 51(4):355–364 20508375 10.1269/jrr.10016

[CR177] Okayasu R (2012) Repair of DNA damage induced by accelerated heavy ions–a mini review. Int J Cancer 130(5):991–1000 21935920 10.1002/ijc.26445

[CR178] Okayasu R et al (2006) Repair of DNA damage induced by accelerated heavy ions in mammalian cells proficient and deficient in the non-homologous end-joining pathway. Radiat Res 165(1):59–67 16392963 10.1667/rr3489.1

[CR179] Paap B, Wilson DM 3rd, Sutherland BM (2008) Human abasic endonuclease action on multilesion abasic clusters: implications for radiation-induced biological damage. Nucleic Acids Res 36(8):2717–2727 18353858 10.1093/nar/gkn118 PMC2377450

[CR180] Paganetti H (2022) Mechanisms and review of clinical evidence of variations in relative biological effectiveness in proton therapy. Int J Radiat Oncol Biol Phys 112(1):222–236 34407443 10.1016/j.ijrobp.2021.08.015 PMC8688199

[CR181] Paganetti H et al (2002) Relative biological effectiveness (RBE) values for proton beam therapy. Int J Radiat Oncol Biol Phys 53(2):407–421 12023146 10.1016/s0360-3016(02)02754-2

[CR182] Pai JT et al (2021) Induction of G2/M Cell cycle arrest via p38/p21(Waf1/Cip1)-dependent signaling pathway activation by bavachinin in non-small-cell lung cancer cells. Molecules 26(17):5161 34500594 10.3390/molecules26175161 PMC8434044

[CR183] Park HA et al (2022) Validation of Genetic Markers Associated with Survival in Colorectal Cancer Patients Treated with Oxaliplatin-Based Chemotherapy. Cancer Epidemiol Biomarkers Prev 31(2):352–361 34862210 10.1158/1055-9965.EPI-21-0814 PMC9789680

[CR184] Patel ZS et al (2020) Red risks for a journey to the red planet: the highest priority human health risks for a mission to Mars. NPJ Microgravity 6(1):33 33298950 10.1038/s41526-020-00124-6 PMC7645687

[CR185] Peitzsch C et al (2019) Cancer stem cells in radiation response: current views and future perspectives in radiation oncology. Int J Radiat Biol 95(7):900–911 30897014 10.1080/09553002.2019.1589023

[CR186] Peng M et al (2018) Opposing roles of FANCJ and HLTF protect forks and restrain replication during stress. Cell Rep 24(12):3251–3261 30232006 10.1016/j.celrep.2018.08.065 PMC6218949

[CR187] Perdyan A et al (2024) Chromosomal positioning and epigenetic architecture influence DNA methylation patterns triggered by galactic cosmic radiation. Sci Rep 14(1):1324 38225252 10.1038/s41598-024-51756-7 PMC10789781

[CR188] Pucci C, Martinelli C, Ciofani G (2019) Innovative approaches for cancer treatment: current perspectives and new challenges. Ecancermedicalscience 13:961 31537986 10.3332/ecancer.2019.961 PMC6753017

[CR189] Ramaekers BL et al (2011) Systematic review and meta-analysis of radiotherapy in various head and neck cancers: comparing photons, carbon-ions and protons. Cancer Treat Rev 37(3):185–201 20817407 10.1016/j.ctrv.2010.08.004

[CR190] Ray S et al (2018) Comparing photon and charged particle therapy using DNA damage biomarkers. Int J Part Ther 5(1):15–24 31773017 10.14338/IJPT-18-00018.1 PMC6871597

[CR191] Roobol SJ et al (2020) Comparison of high- and low-LET radiation-induced DNA double-strand break processing in living cells. Int J Mol Sci 21(18):6602 32917044 10.3390/ijms21186602 PMC7555951

[CR192] Rose M et al (2020) PARP inhibitors: clinical relevance, mechanisms of action and tumor resistance. Front Cell Dev Biol 8:564601 33015058 10.3389/fcell.2020.564601 PMC7509090

[CR193] Ruff SE et al (2020) Roles for MDC1 in cancer development and treatment. DNA Repair (amst) 95:102948 32866776 10.1016/j.dnarep.2020.102948 PMC7669677

[CR194] Russo M et al (2019) Adaptive mutability of colorectal cancers in response to targeted therapies. Science 366(6472):1473–1480 31699882 10.1126/science.aav4474

[CR195] Rycaj K, Tang DG (2014) Cancer stem cells and radioresistance. Int J Radiat Biol 90(8):615–621 24527669 10.3109/09553002.2014.892227 PMC4341971

[CR196] Sadeghi Moghadam M et al (2023) Chromosomal instability in various generations of human mesenchymal stem cells following the therapeutic radiation. Stem Cells Int 2023:9991656 37674788 10.1155/2023/9991656 PMC10480024

[CR197] Sage E, Harrison L (2011) Clustered DNA lesion repair in eukaryotes: relevance to mutagenesis and cell survival. Mutat Res 711(1–2):123–133 21185841 10.1016/j.mrfmmm.2010.12.010 PMC3101299

[CR198] Sage E, Shikazono N (2017) Radiation-induced clustered DNA lesions: repair and mutagenesis. Free Radic Biol Med 107:125–135 27939934 10.1016/j.freeradbiomed.2016.12.008

[CR199] Sai S, Koto M, Yamada S (2023) Basic and translational research on carbon-ion radiobiology. Am J Cancer Res 13(1):1–24 36777517 PMC9906076

[CR200] Saldanha J et al (2023) The adaptive mechanisms and checkpoint responses to a stressed DNA replication fork. Int J Mol Sci 24(13):10488 37445667 10.3390/ijms241310488 PMC10341514

[CR201] Sallmyr A, Tomkinson AE (2018) Repair of DNA double-strand breaks by mammalian alternative end-joining pathways. J Biol Chem 293(27):10536–10546 29530982 10.1074/jbc.TM117.000375 PMC6036210

[CR202] Sarbajna S, Davies D, West SC (2014) Roles of SLX1-SLX4, MUS81-EME1, and GEN1 in avoiding genome instability and mitotic catastrophe. Genes Dev 28(10):1124–1136 24831703 10.1101/gad.238303.114 PMC4035540

[CR203] Sato K, Shimokawa T, Imai T (2019) Difference in acquired radioresistance induction between repeated photon and particle irradiation. Front Oncol 9:1213 31799186 10.3389/fonc.2019.01213 PMC6863406

[CR204] Schilling D et al (2015) Sensitizing tumor cells to radiation by targeting the heat shock response. Cancer Lett 360(2):294–301 25721082 10.1016/j.canlet.2015.02.033

[CR205] Scully R et al (2019) DNA double-strand break repair-pathway choice in somatic mammalian cells. Nat Rev Mol Cell Biol 20(11):698–714 31263220 10.1038/s41580-019-0152-0 PMC7315405

[CR206] Seyedin SN et al (2020) Combination therapy with radiation and PARP inhibition enhances responsiveness to Anti-PD-1 therapy in colorectal tumor models. Int J Radiat Oncol Biol Phys 108(1):81–92 32036006 10.1016/j.ijrobp.2020.01.030 PMC7416439

[CR207] Shiba S et al (2022a) Safety and efficacy of re-irradiation with carbon-ion radiotherapy for pelvic recurrence of rectal cancer after preoperative chemoradiotherapy: a retrospective analysis. In Vivo 36(5):2473–2480 36099090 10.21873/invivo.12983 PMC9463879

[CR208] Shiba S et al (2022b) Long-term survival of two patients with inoperable post-irradiation osteosarcoma treated with carbon-ion radiotherapy: a case report. Radiat Oncol 17(1):68 35379287 10.1186/s13014-022-02040-3 PMC8981945

[CR209] Shiba S et al (2023) Carbon-ion radiotherapy for inoperable solitary fibrous tumor of the skull base: a case report. In Vivo 37(2):908–911 36881080 10.21873/invivo.13161 PMC10026639

[CR210] Shikazono N, Akamatsu K (2020) Strand with mutagenic lesion is preferentially used as a template in the region of a bi-stranded clustered DNA damage site in *Escherichia coli*. Sci Rep 10(1):9737 32546758 10.1038/s41598-020-66651-0 PMC7297740

[CR211] Shikazono N et al (2006) The roles of specific glycosylases in determining the mutagenic consequences of clustered DNA base damage. Nucleic Acids Res 34(13):3722–3730 16893955 10.1093/nar/gkl503 PMC1557791

[CR212] Shimura T (2017) Targeting the AKT/cyclin D1 pathway to overcome intrinsic and acquired radioresistance of tumors for effective radiotherapy. Int J Radiat Biol 93(4):381–385 27910734 10.1080/09553002.2016.1257832

[CR213] Shimura T et al (2011) Targeting the AKT/GSK3beta/cyclin D1/Cdk4 survival signaling pathway for eradication of tumor radioresistance acquired by fractionated radiotherapy. Int J Radiat Oncol Biol Phys 80(2):540–548 21398050 10.1016/j.ijrobp.2010.12.065

[CR214] Shimura T et al (2012) Activation of the AKT/cyclin D1/Cdk4 survival signaling pathway in radioresistant cancer stem cells. Oncogenesis 1(6):e12 23552696 10.1038/oncsis.2012.12 PMC3412645

[CR215] Singh V, Das P (2013) Condensation of DNA–a putative obstruction for repair process in abasic clustered DNA damage. DNA Repair (amst) 12(6):450–457 23582211 10.1016/j.dnarep.2013.03.002

[CR216] Smith J et al (2010) The ATM-Chk2 and ATR-Chk1 pathways in DNA damage signaling and cancer. Adv Cancer Res 108:73–112 21034966 10.1016/B978-0-12-380888-2.00003-0

[CR217] Song B, Yang P, Zhang S (2024) Cell fate regulation governed by p53: Friends or reversible foes in cancer therapy. Cancer Commun (lond) 44(3):297–360 38311377 10.1002/cac2.12520 PMC10958678

[CR218] Srivastava P, Sarma A, Chaturvedi CM (2018) Targeting DNA repair with PNKP inhibition sensitizes radioresistant prostate cancer cells to high LET radiation. PLoS ONE 13(1):e0190516 29320576 10.1371/journal.pone.0190516 PMC5762163

[CR219] Stover EH et al (2016) Biomarkers of response and resistance to DNA repair targeted therapies. Clin Cancer Res 22(23):5651–5660 27678458 10.1158/1078-0432.CCR-16-0247

[CR220] Strigari L et al (2018) Tumour control in ion beam radiotherapy with different ions in the presence of hypoxia: an oxygen enhancement ratio model based on the microdosimetric kinetic model. Phys Med Biol 63(6):065012 28862152 10.1088/1361-6560/aa89ae

[CR221] Suman S et al (2013) Therapeutic and space radiation exposure of mouse brain causes impaired DNA repair response and premature senescence by chronic oxidant production. Aging (albany NY) 5(8):607–622 23928451 10.18632/aging.100587 PMC3796214

[CR222] Suman S et al (2018) The effect of carbon irradiation is associated with greater oxidative stress in mouse intestine and colon relative to gamma-rays. Free Radic Res 52(5):556–567 29544379 10.1080/10715762.2018.1452204

[CR223] Sung H et al (2021) Global cancer statistics 2020: GLOBOCAN estimates of incidence and mortality worldwide for 36 cancers in 185 countries. CA Cancer J Clin 71(3):209–249 33538338 10.3322/caac.21660

[CR224] Tang N et al (2019) Influence of chromatin compaction on simulated early radiation-induced DNA damage using Geant4-DNA. Med Phys 46(3):1501–1511 30689203 10.1002/mp.13405

[CR225] Tian H et al (2015) DNA damage response–a double-edged sword in cancer prevention and cancer therapy. Cancer Lett 358(1):8–16 25528631 10.1016/j.canlet.2014.12.038

[CR226] Timm S et al (2018) Clustered DNA damage concentrated in particle trajectories causes persistent large-scale rearrangements in chromatin architecture. Radiother Oncol 129(3):600–610 30049456 10.1016/j.radonc.2018.07.003

[CR227] Tinganelli W, Durante M (2020) Carbon ion radiobiology. Cancers (basel) 12(10):3022 33080914 10.3390/cancers12103022 PMC7603235

[CR228] Tomimatsu N et al (2017) DNA-damage-induced degradation of EXO1 exonuclease limits DNA end resection to ensure accurate DNA repair. J Biol Chem 292(26):10779–10790 28515316 10.1074/jbc.M116.772475 PMC5491765

[CR229] Tomizawa K et al (2023) Repeated carbon-ion radiation therapy for intrahepatic recurrent hepatocellular carcinoma. Int J Radiat Oncol Biol Phys 116(5):1100–1109 36870514 10.1016/j.ijrobp.2023.02.036

[CR230] Tomsia M et al (2024) Long-term space missions’ effects on the human organism: what we do know and what requires further research. Front Physiol 15:1284644 38415007 10.3389/fphys.2024.1284644 PMC10896920

[CR231] Tonzi P, Huang TT (2019) Role of Y-family translesion DNA polymerases in replication stress: Implications for new cancer therapeutic targets. DNA Repair (amst) 78:20–26 30954011 10.1016/j.dnarep.2019.03.016 PMC6534436

[CR232] Trego KS et al (2016) Non-catalytic roles for XPG with BRCA1 and BRCA2 in homologous recombination and genome stability. Mol Cell 61(4):535–546 26833090 10.1016/j.molcel.2015.12.026 PMC4761302

[CR233] Tsujii H et al (2004) Overview of clinical experiences on carbon ion radiotherapy at NIRS. Radiother Oncol 73(Suppl 2):S41–S49 15971308 10.1016/s0167-8140(04)80012-4

[CR234] Vaitsiankova A et al (2022) PARP inhibition impedes the maturation of nascent DNA strands during DNA replication. Nat Struct Mol Biol 29(4):329–338 35332322 10.1038/s41594-022-00747-1 PMC9010290

[CR235] Venkadakrishnan J et al (2023) Implications of translesion DNA synthesis polymerases on genomic stability and human health. Mol Cell Biol 43(8):401–425 37439479 10.1080/10985549.2023.2224199 PMC10448981

[CR236] Venkhataraman R et al (2001) Enzymatic processing of DNA containing tandem dihydrouracil by endonucleases III and VIII. Nucleic Acids Res 29(2):407–414 11139610 10.1093/nar/29.2.407 PMC29670

[CR237] Vertemara J, Tisi R (2023) Dynamic properties of the DNA damage response Mre11/Rad50 complex. Int J Mol Sci 24(15):12377 37569756 10.3390/ijms241512377 PMC10418313

[CR238] Visnes T et al (2018) Targeting BER enzymes in cancer therapy. DNA Repair (amst) 71:118–126 30228084 10.1016/j.dnarep.2018.08.015

[CR239] Voronina N et al (2020) The landscape of chromothripsis across adult cancer types. Nat Commun 11(1):2320 32385320 10.1038/s41467-020-16134-7 PMC7210959

[CR240] Wang H, Xu X (2017) Microhomology-mediated end joining: new players join the team. Cell Biosci 7:6 28101326 10.1186/s13578-017-0136-8 PMC5237343

[CR241] Wang H et al (2010) Characteristics of DNA-binding proteins determine the biological sensitivity to high-linear energy transfer radiation. Nucleic Acids Res 38(10):3245–3251 20150414 10.1093/nar/gkq069 PMC2879532

[CR242] Wang H et al (2014) Distinct roles of Ape1 protein, an enzyme involved in DNA repair, in high or low linear energy transfer ionizing radiation-induced cell killing. J Biol Chem 289(44):30635–30644 25210033 10.1074/jbc.M114.604959 PMC4215242

[CR243] Wang JS, Wang HJ, Qian HL (2018) Biological effects of radiation on cancer cells. Mil Med Res 5(1):20 29958545 10.1186/s40779-018-0167-4 PMC6026344

[CR244] Watanabe R, Rahmanian S, Nikjoo H (2015) Spectrum of radiation-induced clustered non-DSB damage - a Monte Carlo track structure modeling and calculations. Radiat Res 183(5):525–540 25909147 10.1667/RR13902.1

[CR245] Weber AM, Ryan AJ (2015) ATM and ATR as therapeutic targets in cancer. Pharmacol Ther 149:124–138 25512053 10.1016/j.pharmthera.2014.12.001

[CR246] Whalen MK et al (2008) Specific ATM-mediated phosphorylation dependent on radiation quality. Radiat Res 170(3):353–364 18763865 10.1667/RR1354.1

[CR247] Wilkinson B, Hill MA, Parsons JL (2023) The cellular response to complex DNA damage induced by ionising radiation. Int J Mol Sci 24(5):4920 36902352 10.3390/ijms24054920 PMC10003081

[CR248] Wilson SH, Kunkel TA (2000) Passing the baton in base excision repair. Nat Struct Biol 7(3):176–178 10700268 10.1038/73260

[CR249] Wu Y et al (2015) EEPD1 rescues stressed replication forks and maintains genome stability by promoting end resection and homologous recombination repair. PLoS Genet 11(12):e1005675 26684013 10.1371/journal.pgen.1005675 PMC4684289

[CR250] Wu Y et al (2023a) Molecular mechanisms of tumor resistance to radiotherapy. Mol Cancer 22(1):96 37322433 10.1186/s12943-023-01801-2 PMC10268375

[CR251] Wu W et al (2023b) Mitotic DNA synthesis in response to replication stress requires the sequential action of DNA polymerases zeta and delta in human cells. Nat Commun 14(1):706 36759509 10.1038/s41467-023-35992-5 PMC9911744

[CR252] Wyatt HD et al (2013) Coordinated actions of SLX1-SLX4 and MUS81-EME1 for Holliday junction resolution in human cells. Mol Cell 52(2):234–247 24076221 10.1016/j.molcel.2013.08.035

[CR253] Xie M et al (2020) Bcl2-induced DNA replication stress promotes lung carcinogenesis in response to space radiation. Carcinogenesis 41(11):1565–1575 32157295 10.1093/carcin/bgaa021 PMC8241220

[CR254] Xu X et al (2021) Structure specific DNA recognition by the SLX1-SLX4 endonuclease complex. Nucleic Acids Res 49(13):7740–7752 34181713 10.1093/nar/gkab542 PMC8287910

[CR255] Xue L et al (2009) Regulation of ATM in DNA double strand break repair accounts for the radiosensitivity in human cells exposed to high linear energy transfer ionizing radiation. Mutat Res 670(1–2):15–23 19583974 10.1016/j.mrfmmm.2009.06.016

[CR256] Yajima H et al (2013) The complexity of DNA double strand breaks is a critical factor enhancing end-resection. DNA Repair (amst) 12(11):936–946 24041488 10.1016/j.dnarep.2013.08.009

[CR257] Yang J et al (2018) Salvage carbon ion radiation therapy for locally recurrent or radiation-induced second primary sarcoma of the head and neck. J Cancer 9(12):2215–2223 29937942 10.7150/jca.24313 PMC6010679

[CR258] Yang F, Hu Z, Guo Z (2022a) Small-molecule inhibitors targeting FEN1 for cancer therapy. Biomolecules 12(7):1007 35883563 10.3390/biom12071007 PMC9312813

[CR259] Yang J et al (2022b) Carbon-ion radiotherapy in the treatment of radiation-induced second primary malignancies. Ann Transl Med 10(22):1200 36544688 10.21037/atm-20-200 PMC9761151

[CR260] Yatagai F et al (2002) Heavy-ion-induced mutations in the gpt delta transgenic mouse: effect of p53 gene knockout. Environ Mol Mutagen 40(3):216–225 12355556 10.1002/em.10107

[CR261] Yeo CD et al (2017) The role of hypoxia on the acquisition of epithelial-mesenchymal transition and cancer stemness: a possible link to epigenetic regulation. Korean J Intern Med 32(4):589–599 28704917 10.3904/kjim.2016.302 PMC5511947

[CR262] Yock TI, Caruso PA (2012) Risk of second cancers after photon and proton radiotherapy: a review of the data. Health Phys 103(5):577–585 23032887 10.1097/HP.0b013e3182609ba4

[CR263] Yu F et al (2021) Wnt/beta-catenin signaling in cancers and targeted therapies. Signal Transduct Target Ther 6(1):307 34456337 10.1038/s41392-021-00701-5 PMC8403677

[CR264] Zellweger R et al (2015) Rad51-mediated replication fork reversal is a global response to genotoxic treatments in human cells. J Cell Biol 208(5):563–579 25733714 10.1083/jcb.201406099 PMC4347635

[CR265] Zhang H et al (2015) HIF-1 regulates CD47 expression in breast cancer cells to promote evasion of phagocytosis and maintenance of cancer stem cells. Proc Natl Acad Sci U S A 112(45):E6215–E6223 26512116 10.1073/pnas.1520032112 PMC4653179

[CR266] Zhang J et al (2022a) Flap endonuclease 1 and DNA-PKcs synergistically participate in stabilizing replication fork to encounter replication stress in glioma cells. J Exp Clin Cancer Res 41(1):140 35414100 10.1186/s13046-022-02334-0 PMC9006432

[CR267] Zhang Z et al (2022b) Radiotherapy combined with immunotherapy: the dawn of cancer treatment. Signal Transduct Target Ther 7(1):258 35906199 10.1038/s41392-022-01102-y PMC9338328

[CR268] Zhou Y, Paull TT (2013) DNA-dependent protein kinase regulates DNA end resection in concert with Mre11-Rad50-Nbs1 (MRN) and ataxia telangiectasia-mutated (ATM). J Biol Chem 288(52):37112–37125 24220101 10.1074/jbc.M113.514398 PMC3873567

[CR269] Zhu Y et al (2009) Targeting DNA repair pathways: a novel approach to reduce cancer therapeutic resistance. Cancer Treat Rev 35(7):590–596 19635647 10.1016/j.ctrv.2009.06.005

[CR270] Zou Y et al (2006) Functions of human replication protein A (RPA): from DNA replication to DNA damage and stress responses. J Cell Physiol 208(2):267–273 16523492 10.1002/jcp.20622 PMC3107514

